# Shear stress activates ATOH8 via autocrine VEGF promoting glycolysis dependent-survival of colorectal cancer cells in the circulation

**DOI:** 10.1186/s13046-020-1533-0

**Published:** 2020-01-30

**Authors:** Qiong Huang, Shaowei Li, Xingbin Hu, Mengting Sun, Qijing Wu, Huiru Dai, Yujing Tan, Fei Sun, Chunlin Wang, Xiaoxiang Rong, Wangjun Liao, Jianjun Peng, Jianjun Xiao, Li Huang, Jiao Wang, Bishan Liang, Kelin Lin, Yajing Liu, Min Shi

**Affiliations:** 1 Department of Oncology, Nanfang Hospital, Southern Medical University, Guangzhou, Guangdong 510515 People’s Republic of China; 2 Department of Radiation Oncology, Zhujiang Hospital, Southern Medical University, Guangzhou, Guangdong 510515 People’s Republic of China; 3 Center of Gastrointestinal Surgery, First Affiliated Hospital of Sun Yat-sen University, People’s Republic of China Guangzhou, Guangdong 510080; 4 Department of Chemotherapy, Zhongshan People’s Hospital, Zhongshan, Guangdong 528403 People’s Republic of China; 5 Department of Oncology, the First Affiliated Hospital of Gannan Medical College, Ganzhou, Jiangxi 341000 People’s Republic of China

**Keywords:** Laminar shear stress, Colorectal cancer, ATOH8, Glycolysis, VEGF

## Abstract

**Background:**

Metastasis and recurrence, wherein circulating tumour cells (CTCs) play an important role, are the leading causes of death in colorectal cancer (CRC). Metastasis-initiating CTCs manage to maintain intravascular survival under anoikis, immune attack, and importantly shear stress; however, the underlying mechanisms remain poorly understood.

**Methods:**

In view of the scarcity of CTCs in the bloodstream, suspended colorectal cancer cells were flowed into the cyclic laminar shear stress (LSS) according to previous studies. Then, we detected these suspended cells with a CK8+/CD45−/DAPI+ phenotype and named them mimic circulating tumour cells (m-CTCs) for subsequent CTCs related researches. Quantitative polymerase chain reaction, western blotting, and immunofluorescence were utilised to analyse gene expression change of m-CTCs sensitive to LSS stimulation. Additionally, we examined atonal bHLH transcription factor 8 (ATOH8) expressions in CTCs among 156 CRC patients and mice by fluorescence in situ hybridisation and flow cytometry. The pro-metabolic and pro-survival functions of ATOH8 were determined by glycolysis assay, live/dead cell vitality assay, anoikis assay, and immunohistochemistry. Further, the concrete up-and-down mechanisms of m-CTC survival promotion by ATOH8 were explored.

**Results:**

The m-CTCs actively responded to LSS by triggering the expression of ATOH8, a fluid mechanosensor, with executive roles in intravascular survival and metabolism plasticity. Specifically, ATOH8 was upregulated via activation of VEGFR2/AKT signalling pathway mediated by LSS induced VEGF release. ATOH8 then transcriptionally activated HK2-mediated glycolysis, thus promoting the intravascular survival of colorectal cancer cells in the circulation.

**Conclusions:**

This study elucidates a novel mechanism that an LSS triggered VEGF-VEGFR2-AKT-ATOH8 signal axis mediates m-CTCs survival, thus providing a potential target for the prevention and treatment of hematogenous metastasis in CRC.

## Background

Metastasis is a common cause of death in patients with colorectal cancer (CRC) [1]. Moreover, circulating tumour cells (CTCs) are closely related to tumour metastasis and have become an important biomarker in predicting recurrence and mortality [2]. CTCs survival, and the subsequent adhesion, extravasation, and colonisation of these cells are critical determinants of tumour metastasis [3]. Although most CTCs perish in circulation, facing obstacles including physical stress, anoikis, and immune response [4], approximately 0.1% of CTCs manage to survive as disseminated seeds for eventual relapse [5]. Therefore, exploring the biological characteristics of CTCs and understanding the factors that allow CTCs to survive are beneficial to extinguishing these concealed threats and preventing tumour metastasis.

Living cells continue to perceive and respond to mechanical forces, which are important regulators of cell survival and function [6]. Laminar shear stress (LSS), one of the most crucial mechanical forces, is the friction generated by liquid flowing on the cell surface [7]. Currently, there is sufficient evidence that LSS regulates the survival of various normal cells such as endothelial cells [8], osteoblasts [9], and embryonic stem cells [10]. LSS exerts a lasting influence on CTCs, yet little is known about how LSS is sensed and transduced in CTCs. Some studies reported that LSS could affect the sensitization of TRAIL-mediated tumour cell apoptosis and can also activate MAPK pathway, causing autophagy in hepatocellular carcinoma [11, 12]. However, some distinct views have recently emerged. A study indicated that mechanically sensitive PANX1 channels on the surface of breast cancer cells could respond to LSS stimuli and facilitate the survival of CTCs [13]. Moreover, cancer cells are able to survive pulses of high shear stress, in a lamin A/C dependent manner [14]. Accordingly, more research is needed owing to the scarcity and contradictions in data regarding LSS and CTC survival.

Mechanical transducing molecules, with the capability of sensing and translating a variety of mechanical forces, can transform physical stimulation into biological signals [15]. As a new LSS-response molecule, atonal bHLH transcription factor 8 (ATOH8) is reportedly induced by 10 dyn/cm^2^ LSS in endothelial cells [16]. In addition, ATOH8 is also involved in mechanical factors regulation in multiple biological processes, including angiogenesis [16], skeletal muscle formation [17], and embryonic development [18]. Besides, in previous researches, ATOH8 expression among tumours is heterogeneous, and its role as a tumour suppressor or tumour promoter is still controversial. ATOH8 could inhibit stem cell features of hepatocellular carcinoma cells [19, 20] and EBV-encoded malignant phenotypes of nasopharyngeal carcinoma [21], while promoting cell proliferation and inhibiting apoptosis in CRC cells [22]. In brief, the role of ATOH8 along with its ability to sense LSS in CRC progression deserves further investigation.

Here, starting from the LSS-response molecule, ATOH8, we have unravelled a mechanism by which LSS promotes colorectal cancer cells survival in the circulation and may ultimately lead to hematogenous metastasis.

## Methods

Expanded methods and reagent details are presented in the supplementary materials (Additional file 1: Supplementary methods and materials, Additional file 2: Table S1).

### Patient samples

Peripheral blood samples were collected from 156 CRC patients (clinical cohort 1) with detailed information of blood pressure before any anti-tumour therapy in Nanfang Hospital (Guangzhou, China) from August 2016 to July 2017. CTC isolation and classification were performed as described previously [23]. Details are available in the supplementary materials (Additional file 1: Supplementary methods and materials). Meanwhile, 12 pairs of CRC and adjacent non-tumour tissues (clinical cohort 2) were collected from patients who underwent surgery at Nanfang Hospital between May 2018 and September 2018 to verify the expression of ATOH8. All samples were taken under the approval of the Ethics Committee of Nanfang Hospital along with obtainment of written informed consent from patients.

### Mice

All animal experiments were conducted in accordance with the Public Health Service Policy in Humane Care and Use of Laboratory Animals and were approved by the Ethical Committee of Southern Medical University. BALB/c female nude mice 4–5 weeks of age were purchased from the Experimental Animal Centre, Southern Medical University (Guangzhou, China) and maintained in specific pathogen-free conditions. Subcutaneous tumour and metastatic tumour mouse models were generated as described previously [24]. Details are available in the supplementary materials (Additional file 1: Supplementary methods and materials).

### Cell culture and reagents

The CRC cell lines (LoVo, SW480, SW620, DLD1, HT29, and HCT116) and immortalised intestinal epithelial cell line NCM460 were purchased from Foleibao Biotechnology Development Company (Shanghai, China). Cells were cultured with RPMI 1640 medium with 10% foetal bovine serum (Hyclone, USA) at 37 °C under 5% CO_2_.

### Shear stress experiments

The microfluidic system fabricated by 7 tandem μ-slides I 0.4 (Ibidi, GmbH, Martinsried, Germany) and infusion bump was used to load different levels of shear stress on colorectal cancer cells. Details are available in the supplementary materials (Additional file 1: Supplementary methods and materials).

### Statistical analysis

All data were analysed by SPSS v. 20.0 software (SPSS Inc., Chicago, IL, USA). Results are shown as mean ± SEM from three independent experiments. For comparisons, t-test, Wilcoxon rank-sum test, Chi-squared test, or one-way ANOVA test were used. Kaplan Meier’s method was applied to analyse survival rates. *P* values < 0.05 were considered statistically significant.

## Results

### ATOH8 is a shear stress response molecule and is associated with metastasis and poor prognosis in CRC

CTCs are vital to tumour metastasis, while the number of CTCs is sparse. To solve this research dilemma, previous researchers have used alternative strategies, such as adapted suspension tumour cells or tumour cells suspended and exposed to LSS [25, 26]. Therewith, we simulated the mechanical fluid microenvironment of CTCs using a device that could induce continuous cyclic shear stress on suspended tumour cells and we verified the stability of flow velocity in this flow system, using ANSYS software (Additional file 3: Figure S1a-b). According to previous reports, we set parameters to control LSS within a physiological range of 0–20 dyn/cm^2^ [7]. Most CTCs maintained their original morphology, while some other cells’ edges became indiscernible (Additional file 3: Figure S1c). Importantly, we have identified these suspended colorectal cancer cells with molecular features like CTCs, which are CK8+/CD45−/DAPI+ (Additional file 3: Figure S1d). In conclusion, we defined the above suspension cells exposed to physiological LSS as mimic circulating tumour cells (m-CTCs) and use them as an alternative to CTCs in related experiments in this study.

**Fig. 1:**
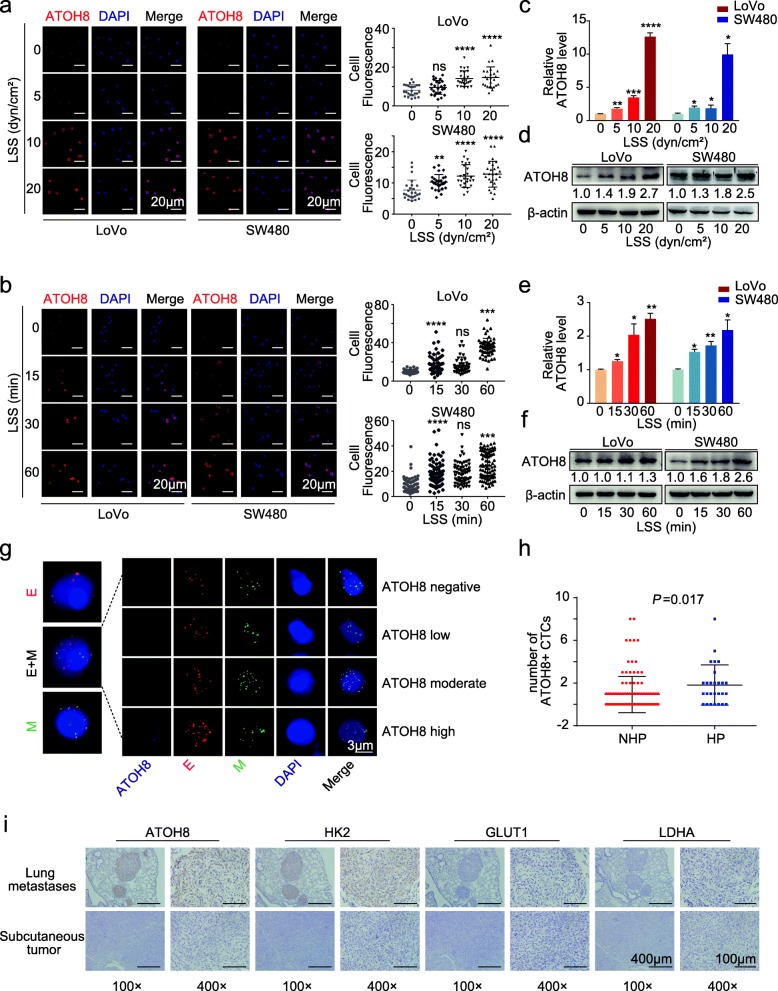
ATOH8 is a shear stress responsive molecule in mimic circulating colorectal cancer cells. **a**, **b** Left, representative immunofluorescence images of ATOH8 expression in suspended LoVo and SW480 cells treated with size gradient (0, 5, 10, 20 dyn/cm^2^; 30 min) (**a**) and time gradient (10 dyn/cm^2^; 0, 15, 30, 60 min) (**b**) laminar shear stress (LSS). Right, quantification of single tumour cell fluorescence intensity in various treatment groups was presented. **c**-**f** Western blotting (WB) and quantitative polymerase chain reaction (qPCR) analysis of ATOH8 expression in suspended LoVo and SW480 cells treated with size gradient (0, 5, 10, 20 dyn/cm^2^; 30 min) (**c**, **d**) and time gradient (10 dyn/cm^2^; 0, 15, 30, 60 min) (**e**, **f**) LSS. **g** Immunofluorescence representation images of different subtypes of CTCs in colorectal cancer patients. Red represents epithelial CTCs, green represents mesenchymal CTC, red and green represent mixed CTC, and purple shade represents different expression levels of ATOH8 in CTCs. **h** In colorectal cancer patients with hypertension, the number of ATOH8 (+) CTCs (CTCs with ATOH8 expression, including ATOH8 low, moderate and high expression) were higher. **i** Representative immunohistochemistry images of ATOH8, HK2, GLUT1 and LDHA expression in serial sections of mouse subcutaneous tumour tissue and colon cancer lung metastases. *P < 0.05, **P < 0.01, ***P < 0.001 and ****P < 0.0001

Firstly, LoVo and SW480 suspended cells were loaded into the shear stress device, and the expression of ATOH8, an LSS response molecule was detected. After size-gradient and time-gradient shear stress stimulation, the results of immunofluorescence analysis, quantitative polymerase chain reaction (qPCR) and western blotting (WB) were concurrent, implying that the expression levels and the nuclear localisation of ATOH8 were increased in CRC m-CTCs (Fig. 1a-f). The mRNA level of ATOH8 in CRC m-CTCs increased obviously after 15 min of LSS and reached a maximum at around 4 h (Fig. 1e, Additional file 3: Figure S1e).

To further investigate changes of ATOH8 in response to LSS in vivo, we harvested peripheral blood samples from clinical cohort 1 and carried out CTC assessment. A previous study has reported that patients with hypertension often have high blood LSS levels [27]. Therefore, after excluding patients receiving antihypertensive therapy (*n* = 15), we divided patients into hypertension (HP, *n* = 27) and non-hypertension (NHP, *n* = 114) groups according to their previous history of hypertension. Other baseline clinical characteristics of the two groups were compared and no significant differences were detected (Additional file 4: Table S2). In both groups, CTCs with three different phenotypes (epithelial phenotype, mixed epithelial/mesenchymal phenotype, and mesenchymal phenotype) and different ATOH8 expression levels were observed and enumerated (Fig. 1g). As predicted, the proportion of CRC patients with a total number of CTCs ≥5 cells/5 mL was higher in the HP group (Additional file 3: Figure S1f-g). Moreover, the total number of ATOH8 (+) CTCs increased in the HP group (Fig. 1h). Together, these data indicate that ATOH8 expression in CRC CTC is sensitive to LSS.

Additionally, ATOH8 expression was further assessed in samples from clinical cohort 2 via WB, revealing that ATOH8 was markedly upregulated in tumour tissues relative to adjacent normal tissues (ANTs) (Additional file 3: Figure S2a). ATOH8 expression levels were also higher in CRC cell lines than in NCM460 (Additional file 3: Figure S2b). Next, Kaplan-Meier analysis was conducted, revealing a significant correlation between ATOH8 upregulation and poor overall survival (OS) (*P* = 0.0335, TCGA) (Additional file 3: Figure S2c). Together, these results indicate that ATOH8 is upregulated in CRC tissues and may predict a poor prognosis.

Furthermore, we quantified ATOH8 expression levels in another cohort including 333 primary and 167 metastatic colorectal tumours, and *ATOH8* was upregulated in metastatic CRC tissues (*P* < 0.0001, GSE131418) (Additional file 3: Figure S2d). We then established mouse subcutaneous and metastatic tumour models (Additional file 3: Figure S2e) and used serial sections, and immunohistochemical (IHC) staining to confirm ATOH8 upregulation in metastatic tumours in comparison with that in primary tumours (Fig. 1i, Additional file 3: Figure S2f). The results suggested that ATOH8 upregulation in CRC cells may happen during the “CTC stage” and be associated with tumour metastasis. Additionally, Kaplan-Meier analysis of progression-free survival (PFS) in 153 surgically treated patients with stage II-III colorectal cancer from GSE103479 was conducted according to the expression of the ATOH8, revealing a significant correlation between ATOH8 upregulation and poor PFS (*P* = 0.0169, GSE103479) (Additional file 3: Figure S2 g). And unexpectedly, in the clinical cohort 1, we found that the proportion of ATOH8 (+) CTCs was higher in the subgroup with high metastatic mesenchymal CTCs or a total CTC number ≥ 5 cells/5 mL (HP group) (Additional file 3: Figure S2 h), suggesting that ATOH8 (+) CTCs are potentially associated with a high risk of metastasis.

In summary, LSS can trigger the ATOH8 upregulation in m-CTCs, which may affect hematogenous metastasis and prognosis of colorectal cancer.

### ATOH8 elevation in m-CTCs enables intravascular survival and provides advantages in hematogenous metastasis

**Fig. 2:**
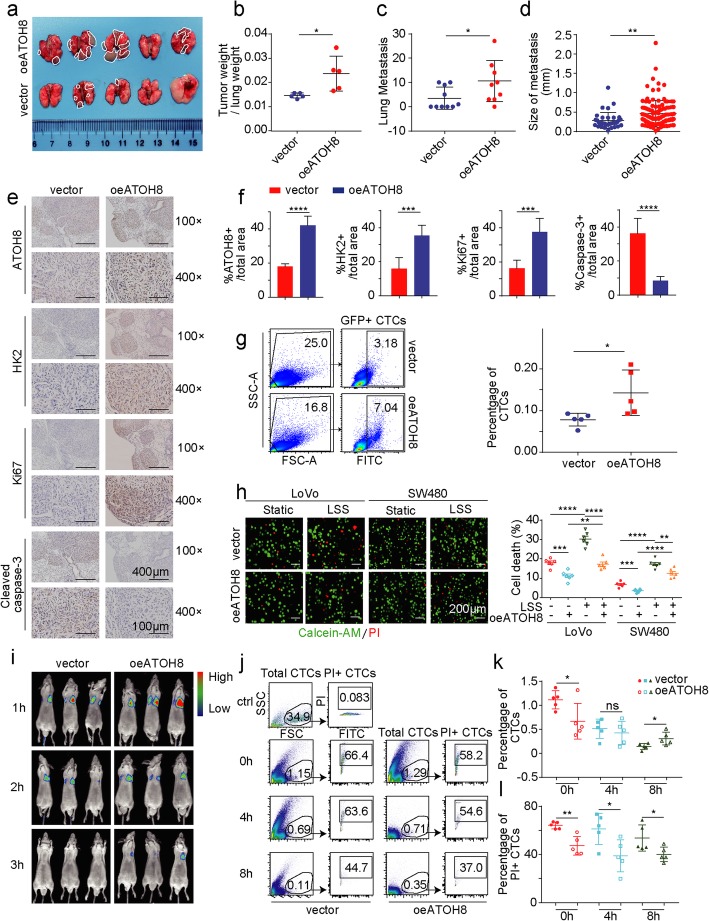
ATOH8-overexpressing colorectal tumour cells tend to survive and metastasize in the circulation. **a** Stably transfected SW480 cells with GFP labelling were injected intravenously into nude mice and then lung metastasis model was established 4 weeks later. The gross view of lung metastasis from nude mice in vector or ATOH8-overexpressing groups was presented. **b** The statistical result of the weight rate of lung metastasis/ lung tissue in vector or ATOH8-overexpressing groups. **c**, **d** The statistical result of metastatic nodule numbers (**c**) and sizes (**d**) in the lungs from vector or ATOH8-overexpressing groups. **e, f** Immunohistochemistry (**e**) and quantification (**f**) graph of the ratio of ATOH8+, HK2+, Ki67+ and cleaved caspase 3+ cells of tumour samples from the ATOH8 overexpression group and control groups. **g** Left, the GFP (+) SW480 cancer cells percentage in the blood of lung metastatic nude mice was analysed by flow cytometry. Right, the statistical result of the percentage of GFP (+) SW480 was presented. **h** Live/dead cell vitality assay of suspended LoVo and SW480 cells were treated with LSS (10 dyn/cm^2^, 30 min). Representative fluorescence images (Left) and quantification of dead cells (Right) were displayed. Red in the images denotes dead cells, while green denotes live cells. **i** Vector or ATOH8-overexpressing SW480 cells with luciferase were injected intravenously, and in vivo imaging was performed at 1, 2 and 3 h after the injection. **j** Vector or ATOH8-overexpressing SW480 cells with GFP labelling were injected intravenously, and flow cytometric cell apoptosis assays was performed at 0, 4 and 8 h after the injection. Different groups of representative flow cytometry diagrams were displayed. **k, l** The statistical result of the number of total CTCs (**k**) and apoptotic CTCs (PI+ CTCs, **l**) based on **j**. *P < 0.05, **P < 0.01, ***P < 0.001 and ****P < 0.0001

Using a mouse model of lung metastasis (Additional file 3: Figure S3a), we found that ATOH8 overexpression markedly increased tumour volume, tumour weight and metastatic foci in the lungs of nude mice, as expected (Fig. 2a-b, Additional file 3: Figure S3b). Furthermore, haematoxylin and eosin staining revealed a sharp increase in the rate of pulmonary metastasis in mice with ATOH8 overexpression (Fig. 2c-d, Additional file 3: Figure S3c). IHC staining of tumours indicated that ATOH8 and Ki-67 were upregulated upon ATOH8 overexpression, while cleaved caspase-3, an apoptotic marker, was downregulated (Fig. 2e-f). Interestingly, we found a significant increase in the number of CTCs in peripheral blood of mice in the ATOH8 overexpression group (Fig. 2g), which means that the increased lung metastasis after overexpression of ATOH8 may be related to the elevated CTC number.

Previous studies have reported that the number of CTCs is an independent predictor of PFS and OS in patients with metastatic colorectal cancer [28]. However, metastatic colonization is a highly inefficient process wherein most CTCs die, and the survived CTCs are rare [4]; therefore, it is important to identify the reason for upregulated CTC number in the ATOH8-overexpressing group of mice. Our further experiments found that ATOH8 efficiently promoted the migratory and invasive abilities (Additional file 3: Figure S4a-b), respectively. Furthermore, the effect of suppression of ATOH8 on apoptosis was detected by MTT and flow cytometry in suspended LoVo and SW480 cells, while cell cycle didn’t change significantly in ATOH8-overexpression group (Additional file 3: Figure S4c-e). Additionally, the qPCR results indicated that the anoikis markers *N-cadherin*, *Vimentin* and *Laminin5* were increased after ATOH8 overexpressing in LoVo and SW480 cells, while E-cadherin is decreased (Additional file 3: Figure S4f). The data above suggested that ATOH8 overexpressing may increase the number of CTCs by inhibiting death rather than promoting proliferation. To monitor cell death in real time and reduce additional LSS interference, we added live/dead cell dye into the culture of m-CTCs, and the experiment determined that overexpression of ATOH8 in CRC m-CTCs leads to a decreased cell death rate (Fig. 2h). Furthermore, we aimed to examine the survival advantage of ATOH8-overexpressing CTCs in vivo. Thus, vector or ATOH8-overexpressing SW480 cells with luciferase were intravenously injected into nude mice (Additional file 3: Figure S3a). Whole-body imaging indicated that ATOH8 overexpression decelerated the reduction in CTCs (Fig. 2i). Moreover, tracking of GFP-labelled SW480 cells in mice revealed that CTCs were extremely rare in blood, with the ratio fluctuating between 0.1 and 1.42% (Fig. 2j-k, Additional file 3: Figure S3a). As shown in Fig. 2l, the percentage of CTCs undergoing cell death (PI-positive CTCs) gradually decreased in the ATOH8-overexpressing group, with a reduction of approximately 10–20%.

Concisely, these in vitro and in vivo experimental data suggest that ATOH8-overexpressing m-CTCs have a distinctive capacity to resist death and exert their vital effects in CRC metastasis.

### ATOH8 promotes CRC m-CTCs survival via HK2-mediated glycolysis

**Fig. 3:**
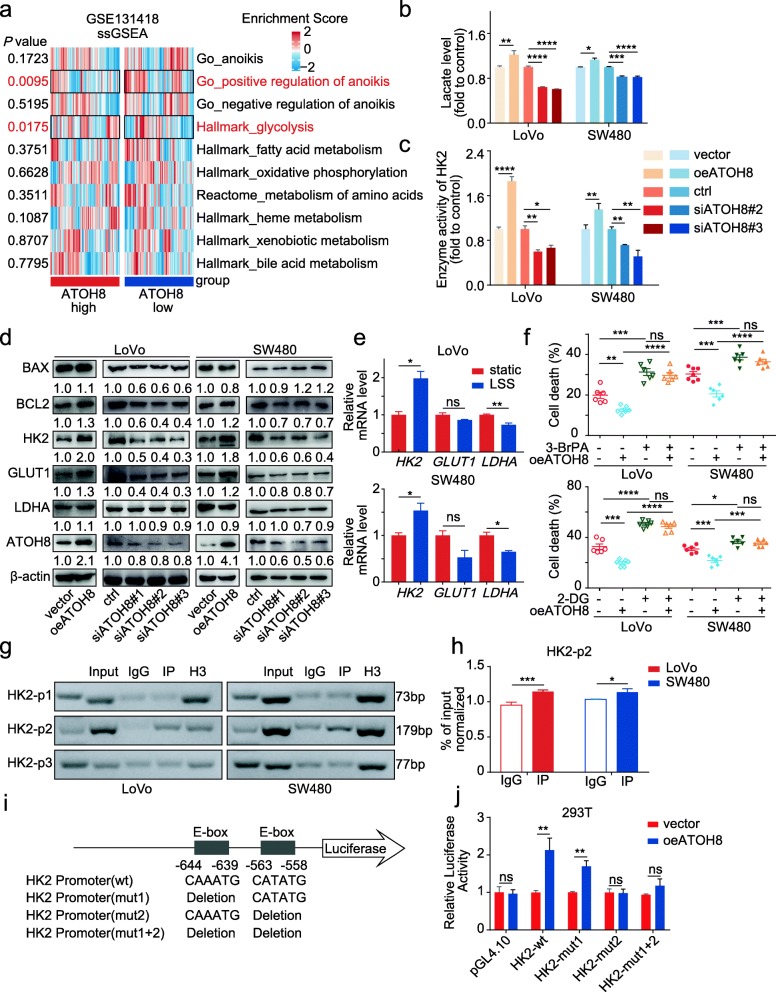
ATOH8 induced intravascular survival via HK2-mediated glycolysis. **a** Single sample gene set enrichment analysis (ssGSEA) of gene-containing signature in ATOH8_high_ and ATOH8_low_ group in the colorectal cancer metastasis cohort from GSE131418 and the results of anoikis-related and key metabolic pathways were presented. **b**, **c** Overexpression of ATOH8 promoted lactate production (**b**) and HK2 enzyme activity (**c**) in suspended LoVo and SW480 cells, while opposite effects were observed when silencing ATOH8. **d** WB analysis of the expression level of glycolytic enzymes HK2, LDHA, and GLUT1 and apoptotic markers BAX, BCL2 in suspended LoVo and SW480 cells after overexpressing or silencing ATOH8. **e** qPCR analysis of *HK2*, *LDHA*, and *GLUT1* expression in suspended LoVo and SW480 cells treated with LSS (10 dyn/cm^2^, 30 min). **f** Live/dead cell vitality assay for cell death rate in LoVo and SW480 mimic circulating tumour cells (m-CTCs) after overexpressing ATOH8 and treating with or without 1 mM 2-Deoxy-D-glucose (2-DG) or 2 nM 3-bromopyruvate (3-BrPA) (10 dyn/cm^2^, 30 min). **g** LoVo and SW480 cells were transfected with flag tagged ATOH8 and harvested for a chromatin immunoprecipitation (ChIP) assay to detect the enrichment of ATOH8 around the HK2 promoter. PCR products amplified with the indicated primers using anti-Flag antibody immunoprecipitated DNA (IP) as a template and anti-IgG or anti-histone H3 antibody immunoprecipitated DNA as negative or positive control. **h** Quantity of ChIP DNA pulled down. **i** The promoter of HK2 contains ATOH8 binding domains, and the binding sites of HK2 promoter wild-type or mutation vector were displayed. **j** Luciferase activity in 293 T cells when ATOH8 wild-type vector was co-transfected with HK2 promoter wild-type or mutation vector. *P < 0.05, **P < 0.01, ***P < 0.001 and ****P < 0.0001

To evaluate the potential mechanisms underlying the pro-survival effects of ATOH8 in m-CTCs, single sample Gene Set Enrichment analysis (ssGSEA) were performed in the metastatic colorectal cancer cohort from GSE131418 (Fig. 3a, Additional file 3: Figure S5a, Additional file 5: Table S3). The results indicated that the gene set of positive regulation of anoikis could be enriched in ATOH8_low_ group (Fig. 3a), supporting our hypothesis that ATOH8_high_ CTCs is prone to survival in the circulation. On the other hand, our previous studies have found that metabolic reprogramming is a key factor mediating the anoikis resistance of tumour cells [24]. Further, to explore the association between metabolism and the ATOH8 mediated CTC survival, ssGSEA analyses were performed, and the data revealed that only glycolysis, a vital metabolic pathway in tumour cells, was significantly different between ATOH8_high_ and ATOH8_low_, rather than fatty acid metabolism, oxidative phosphorylation and amino acid metabolism (Fig. 3a), etc. A previous study has reported that activated glycolysis was closely associated with anoikis tolerance and cell survival in prostate cancer [29]. So, we hypothesised that activated glycolysis may be related to the pro-survival potential of ATOH8.

In fact, the present results confirmed that ATOH8 accelerates glucose uptake, via a 2-NBDG assay (Additional file 3: Figure S5b). Moreover, HK2 enzyme activity and both ATP and lactate production were induced via ATOH8 overexpression, while the opposite result was observed upon silencing ATOH8 (Fig. 3b-c, Additional file 3: Figure S5c). In short, ATOH8 did activate glycolysis in suspended CRC cells. Additionally, a significant positive correlation was observed between *ATOH8* and the key glycolytic enzymes *HK2*, *GLUT1* (Additional file 3: Figure S5d, Additional file 6: Table S4). To elucidate the molecular mechanism underlying ATOH8-induced glycolysis, we screened the expression of HK2, GLUT1, and LDHA on both transcriptional and translational level in ATOH8-overexpressing or -silenced suspended CRC cells. Our results showed that ATOH8 significantly upregulated glycolytic factors *HK2* and *GLUT1* at the mRNA level, rather than *LDHA* and *MCT1* (Additional file 3: Figure S5e). Further, among the candidate factors, only HK2 showed an enhanced expression at the protein level (Fig. 3d), concurrent with the tissue IHC analysis mentioned above (Fig. 1i, Fig. 2e). More importantly, *HK2* was also increased in tumour cells under LSS independent of ATOH8 overexpression (Fig. 3e). These results indicate that ATOH8 probably maintains CTC survival by promoting HK2.

Reduced ROS production and mitochondrial-associated HK2 inhibit glycolysis-mediated cell survival [30]. Our results showed that ATOH8 overexpression decreased ROS accumulation and potentially promoted mitochondrial localisation of HK2 binding to mitochondrial VDAC, contributing to cell survival (Additional file 3: Figure S6a-b). Furthermore, functional experiments revealed that promotion of glucose uptake, HK2 enzyme activity, and ATP and lactate production by ATOH8 overexpression was partially recovered after using HK2 inhibitors (2-DG and 3-BrPA) in suspended CRC cells (Additional file 3: Figure S6c-f). And as expected, in the live/dead cell vitality assay, HK2 inhibitors almost completely reversed the ATOH8-induced m-CTC survival (Fig. 3f). These results further corroborate the pro-survival function of ATOH8 in CRC m-CTCs by upregulating HK2.

ATOH8 is a bHLH domain transcription factor that binds to E-box sequences and activates transcription [19]. To determine whether there is a direct regulation relationship between ATOH8 and HK2, we designed three different sets of primers around the TSS (− 1000 to + 1 bp). In ATOH8-overexpressing CRC cells, ChIP-qPCR data revealed that binding of ATOH8 to DNA fragment 2 (HK2-p2, nt − 702 and nt − 524) was increased, with no significant enrichment at DNA fragments 1 (HK2-p1, nt − 866 and nt − 794) and 3 (HK2-p3, nt − 222 and nt − 145) (Fig. 3g-h). The HK2 promoter region contains two E-Box sequences (both in DNA fragment 2), as predicted using Genomatix (https://www.genomatix.de/, Fig. 3i). To further analyse the exact binding sites of ATOH8 on the HK2 promoter, we designed five plasmids for the HK2 promoter region, which are pGL4.10, pGL4.10-HK2-wt, pGL4.10-HK2-mut1, pGL4.10-HK2-mut2, pGL4.10-HK2-mut1 + 2 (Fig. 3i). Next, we conducted a dual luciferase reporter assay and the results showed that the luciferase activities of pGL4.10-HK2-wt and pGL4.10-HK2-mut1 were significantly increased in ATOH8-overexpressing 293 T cells, but not pGL4.10-HK2-mut2 and pGL4.10-HK2-mut1 + 2, suggesting that the E-box site (nt − 563 and nt − 558, CATATG) is essential for ATOH8-induced HK2 promoter activation (Fig. 3j). Together, our data show that ATOH8 promotes CTC survival by binding to HK2 and directly increasing its transcriptional activity.

### LSS-induced autocrine VEGF participates in ATOH8-mediated CRC m-CTCs survival

**Fig. 4:**
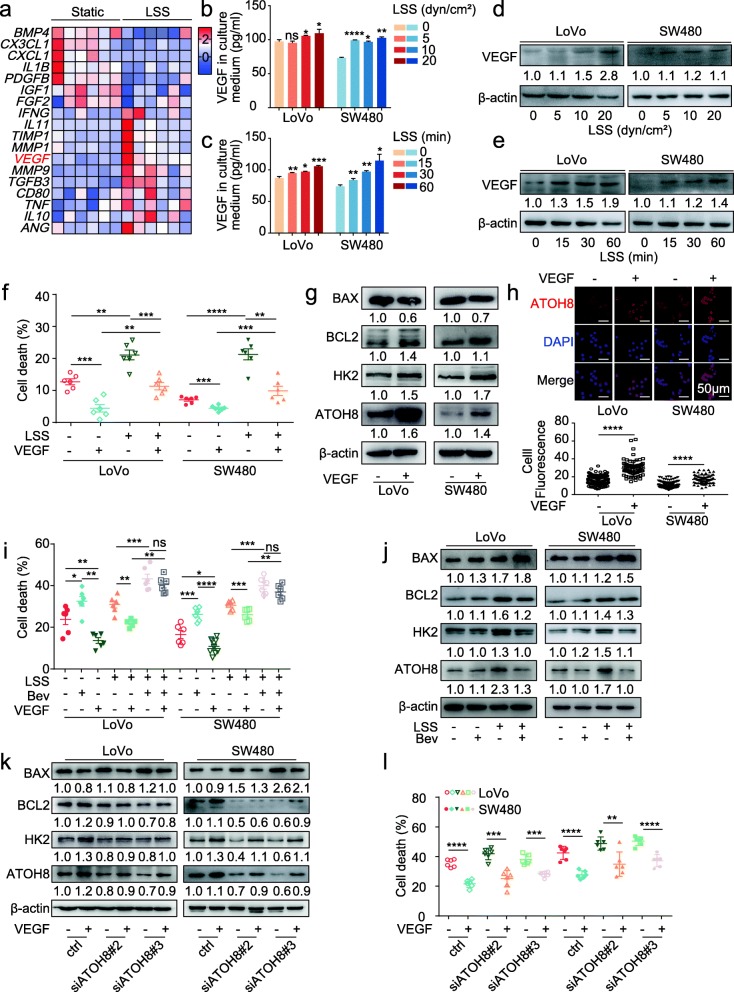
Increased VEGF autocrine is responsible for ATOH8 upregulation in a shear stress environment. **a** Heat map of aberrantly expressed cytokines and cytokine receptors in endothelial cells undergoing LSS (6 dyn/cm^2^, 24 h) from GSE52211. **b, c** Enzyme-linked immunosorbent assay (ELISA) measurement of human VEGF protein levels in LoVo and SW480 m-CTCs medium, treated with size gradient (0, 5, 10, 20 dyn/cm^2^; 30 min) (**b**) and time gradient (10 dyn/cm^2^; 0, 15, 30, 60 min) (**c**) LSS. **d, e** WB analysis of VEGF expression in LoVo and SW480 m-CTCs treated with size gradient (0, 5, 10, 20 dyn/cm^2^; 30 min) (**d**) and time gradient (10 dyn/cm^2^, 0, 15, 30; 60 min) (**e**) LSS. **f** Live/dead cell vitality assay for cell death rate in suspended LoVo and SW480 cells treated with or without LSS (10 dyn/cm^2^, 30 min) and VEGF (10 ng/mL). **g** WB analysis of expression level of ATOH8, HK2, BAX and BCL2 in suspended LoVo and SW480 cells treated with 10 ng/mL VEGF for 24 h. **h** Upper, representative immunofluorescence images of ATOH8 expression in suspended LoVo and SW480 cells treated with 10 ng/mL VEGF for 24 h. Down, quantification of fluorescence intensity. **i** Live/dead cell vitality assay in suspended LoVo and SW480 cells treated with LSS (10 dyn/cm^2^, 30 min), with or without 10 ng/mL VEGF and 5 μg/ mL bevacizumab. **j** WB analysis of expression level of ATOH8, HK2, BAX and BCL2 in suspended LoVo and SW480 cells treated with or without LSS (10 dyn/cm^2^, 30 min) and with or without 5 μg/ mL bevacizumab. **k** Suspended LoVo and SW480 cells transfected with ctrl or si-ATOH8 were seeded in low attachment 6-well plate and treated with 10 ng/mL VEGF for 24 h, and the expression of ATOH8, HK2, BAX and BCL2 were performed. **l** Live/dead cell vitality assay for cell death rate in suspended LoVo and SW480 cells transfected with ctrl or si-ATOH8 and then treated with or without VEGF (10 ng/mL). *P < 0.05, **P < 0.01, ***P < 0.001 and ****P < 0.0001

Cellular plasticity is important for understanding tumorigenesis and tumour progression [31]. Accordingly, we observed that the molecular plasticity of ATOH8 driven by LSS facilitated m-CTC survival. Emerging evidence has revealed that cytokine secretion contributes to LSS-related mechanotransduction [32]. Furthermore, bioinformatics analysis revealed that *VEGF* is secreted by LSS-stimulated endothelial cells (GSE13712 and GSE52211) (Fig. 4a, Additional file 3: Figure S7a, Additional file 7: Table S5). We investigated cytokine and cytokine receptor levels in CRC cells and found that LSS notably upregulated *VEGF* (Additional file 3: Figure S7b). Consistent with ATOH8, VEGF upregulation and increased secretion were observed in both CRC m-CTCs through gradual increases in the intensity and duration of LSS (Fig. 4b-e, Additional file 3: Figure S7c). It is worth noting that previous literature has shown that when human exfoliated deciduous teeth were exposed to 4 dyn/cm^2^ for about 4 h, VEGF secretion gradually reached platform phase [33], which is similar to the trend of ATOH8 mRNA in CRC m-CTCs (Additional file 3: Figure S1e).

VEGF, a key cytokine mainly secreted by endothelial cells and tumour cells, reportedly promotes angiogenesis, activates glycolysis, and induces anoikis tolerance [34]. Indeed, in VEGF-treated suspended CRC cells, we detected less ROS accumulation and anoikis (Additional file 3: Figure S7d-e). Meanwhile, VEGF improved the cell viability of CRC m-CTCs CTCs, either evaluated by live or dead cell assay (Fig. 4f). These results suggest that autocrine VEGF secretion from CRC m-CTCs may contributes to LSS-induced ATOH8 upregulation and changes in cell survival.

To examine this assumption, we measured ATOH8, HK2, BCL2, and BAX expression levels in LoVo and SW480 cell suspensions cultured in media supplemented with VEGF for 24 h (Fig. 4g, Additional file 3: Figure S7f). As predicted, VEGF upregulated ATOH8, HK2, and BCL2/BAX ratio in CRC cells. Especially, increased nuclear translocation of ATOH8 and HK2 enzyme activity was observed in VEGF-treated suspended CRC cells (Fig. 4hg, Additional file 3: Figure S7 g). These results indicated that VEGF could promote ATOH8 expression and activate downstream glycolysis. In addition, Bevacizumab, a humanised mouse anti-human VEGF antibody, could inhibit the CRC m-CTCs survival mediated by VEGF (Fig. 4i) and block the LSS-induced ATOH8 and HK2 upregulation (Fig. 4j), implying the ATOH8 upregulation induced by LSS is related to the secretion of VEGF.

Furthermore, as the rescue experiments shown, siATOH8 partially reversed VEGF-induced upregulation of HK2 activity and ATOH8, HK2, and BCL2/BAX ratio (Fig. 4k, Additional file 3: Figure S7 h) and restored VEGF-induced reduction of ROS production and anoikis (Additional file 3: Figure S7i-j) in suspended CRC cells. And ATOH8 suppression by siRNA partially reversed the pro-survival phenotype of CRC m-CTCs owing to VEGF stimulation (Fig. 4l). Together, these findings suggest that the promotion of CRC m-CTC survival mediated by ATOH8 partially depends on LSS-induced autocrine VEGF signalling.

### The VEGF-VEGFR2 modulates ATOH8 via AKT signalling pathway to sustain CRC m-CTCs survival

**Fig. 5:**
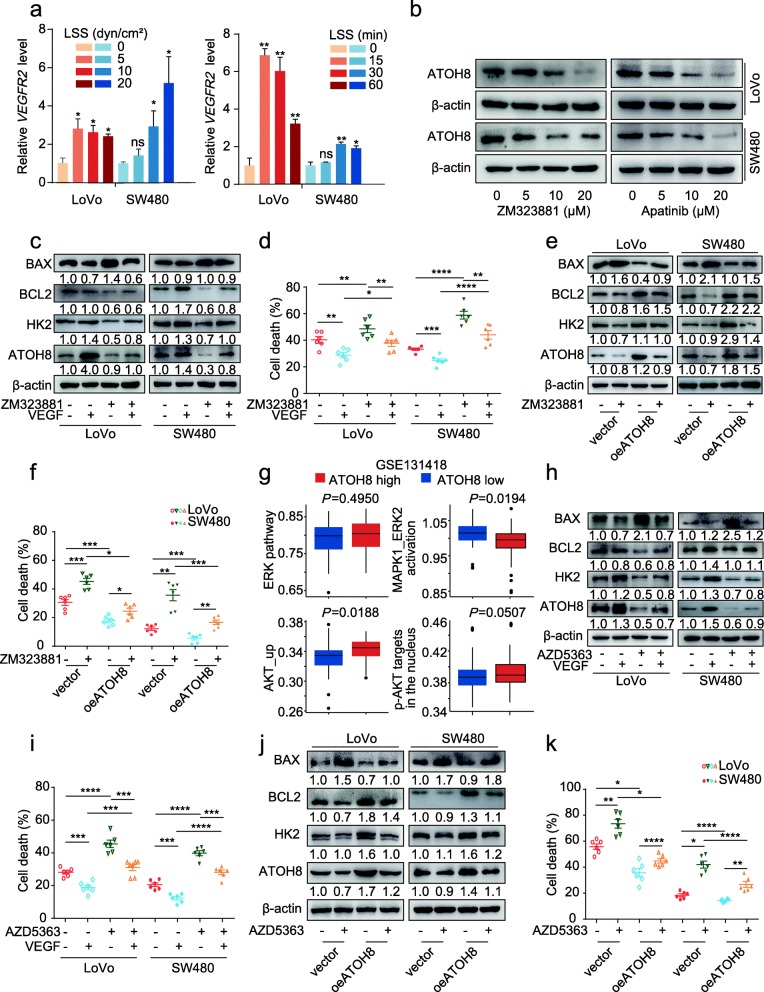
VEGFR2-AKT pathway contributes to VEGF induced ATOH8 expression in CRC m-CTCs. **a** The qPCR analysis of *VEGFR2* expression in suspended LoVo and SW480 cells treated with size gradient (0, 5, 10, 20 dyn/cm^2^, 30 min, Left) and time gradient (10 dyn/cm^2^, 0, 15, 30, 60 min, Right) LSS. **b** Suspended LoVo and SW480 cells was treated with VEGFR2 inhibitors ZM323881 (0, 5, 10, 20 μM) or Apatinib (0, 5, 10, 20 μM), and the relative change in ATOH8 expression was analysed by WB. **c, d** Suspended LoVo and SW480 cells treated with or without 10 ng/mL VEGF and with or without 10 μM VEGFR2 inhibitor (ZM323881). The expression of ATOH8, HK2, BAX and BCL2 were analysed (**c**), additionally, cell death was tested by Live/dead cell vitality assay (10 dyn/cm^2^, 30 min) (**d**). **e, f** Suspended LoVo and SW480 cells with ATOH8 overexpression were treated with or without 10 μM VEGFR2 inhibitor (ZM323881). The expression of ATOH8, HK2, BAX and BCL2 (**e**) and the cell death (**f**) were examined by WB and live/dead cell vitality assay (10 dyn/cm^2^, 30 min) separately. **g** The ssGSEA of AKT or ERK signalling pathways in ATOH8_high_ and ATOH8_low_ group in the colorectal cancer metastasis cohort from GSE131418. **h, i** Suspended LoVo and SW480 cells treated with or without 10 ng/mL VEGF and with or without 10 μM AKT inhibitor (AZD5363). The protein levels of ATOH8, HK2, BAX and BCL2 were analysed by WB (**h**), additionally, cell death was tested by Live/dead cell vitality assay (10 dyn/cm^2^, 30 min) (**i**). **j** WB analysis of expression level of ATOH8, HK2, BAX and BCL2 detected in suspended LoVo and SW480 cells with ATOH8 overexpression, with or without 10 μM AKT inhibitor (AZD5363) for 24 h. **k** Live/dead cell vitality assay in suspended LoVo and SW480 cells after ATOH8 overexpression treated with LSS (10 dyn/cm^2^, 30 min), with or without 10 μM AKT inhibitor (AZD5363). *P < 0.05, **P < 0.01, ***P < 0.001 and ****P < 0.0001

VEGF exerts its effects by binding to VEGF receptor 2 (VEGFR2) in CRC cells [35]; similarly, the present results show that *VEGFR2* was upregulated in CRC m-CTCs exposed to LSS (Fig. 5a). Moreover, VEGFR2 inhibitor ZM323881 and Apatinib markedly downregulated ATOH8 in CRC cell suspensions (Fig. 5b). Then, we investigated whether VEGFR2 regulates VEGF-ATOH8 signalling in CRC m-CTCs. Indeed, blocking of VEGFR2 signals partially reversed HK2 activity upregulation and ATOH8, HK2, and BCL2/BAX expression induced by VEGF in suspended CRC cells (Fig. 5c, Additional file 3: Figure S8a). Furthermore, the VEGF-induced reduction in cellular ROS levels and CTC death of CRC m-CTCs was partly restored upon treatment with VEGFR2 inhibitor (Fig. 5d, Additional file 3: Figure S8b). As predicted, ATOH8 overexpression partially reversed the downregulation of ATOH8, HK2, and BCL2/BAX ratio via inhibition of VEGFR2 signals (Fig. 5e) and CTC death induced by ZM323881 (Fig. 5f). Taken together, these data suggest that VEGFR2 is relatively responsible for VEGF-mediated ATOH8 upregulation in CRC cells.

**Fig. 6:**
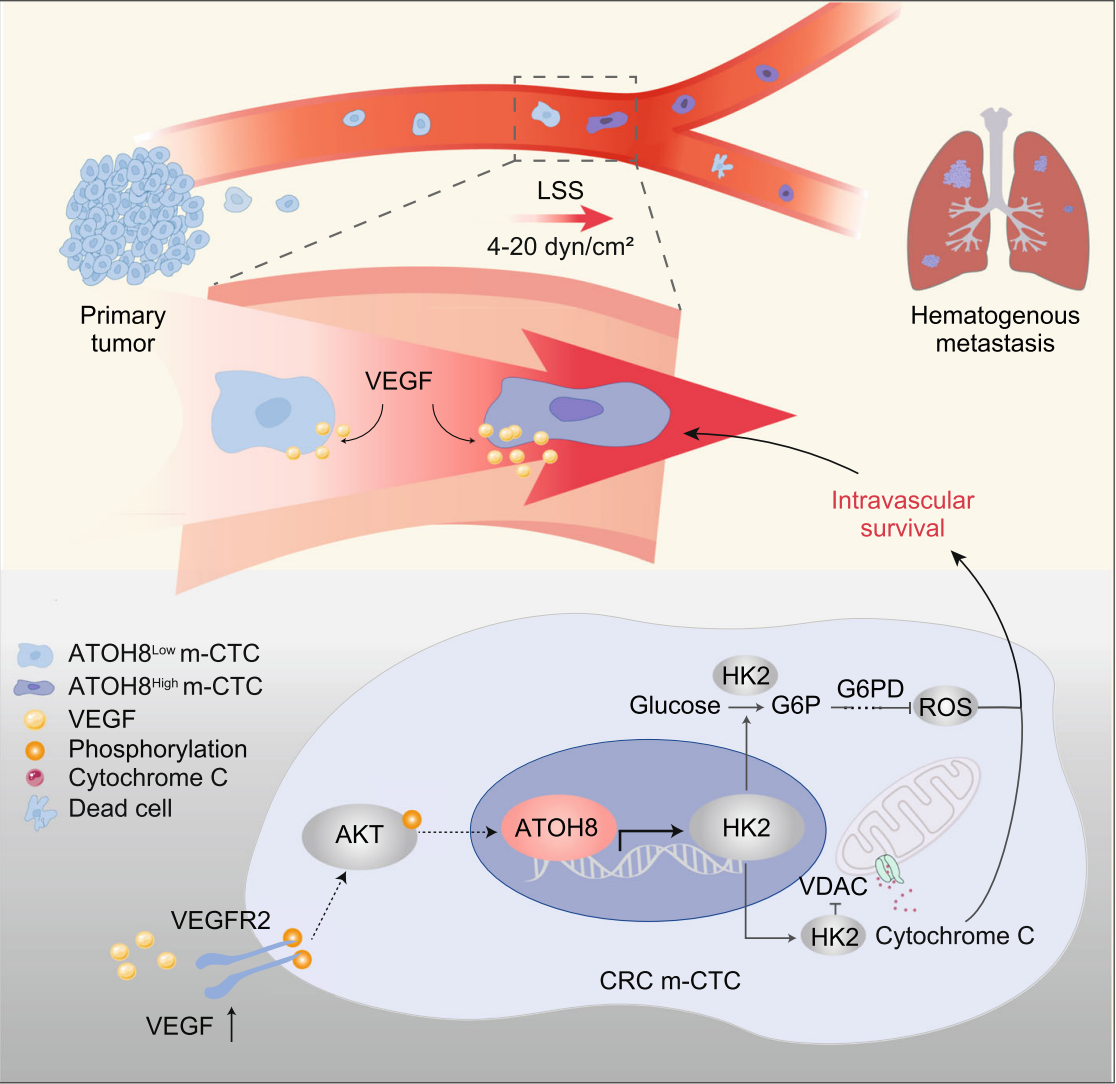
Schematic representation of the LSS-triggered VEGF-ATOH8 signal axis mediates CRC m-CTCs survival. The exposure of 4–20 dyn/cm^2^ LSS can upregulate ATOH8 expression by promoting autocrine VEGF secretion of colorectal cancer cells in the circulation. These ATOH8_high_ m-CTCs possess an advantage in surviving and establishing distant metastases. Mechanically, in CRC m-CTCs responding to LSS stimulation, VEGF activates the downstream AKT pathway by acting on the VEGFR2 receptor, thereby facilitating ATOH8 expression. Meanwhile, ATOH8 advances HK2 transcription, which not only enhances the mitochondrial binding of HK2 to VDAC, but also inhibits ROS production through activating glycolysis. Both ultimately contribute to the survival of CRC m-CTCs

Moreover, the AKT and ERK signalling pathways, both of which are downstream of VEGFR2, were reportedly associated with cell survival [36, 37]. However, it remains unclear whether AKT or ERK signalling is responsible for CTC survival induced by the VEGF/VEGFR2/ATOH8 axis. We found that the treatment of CRC cell suspensions with AKT inhibitors (AZD5363 and MK-2206), rather than ERK inhibitor (SCH772984), downregulated ATOH8 (Additional file 3: Figure S8c-e). Consistent with this, the results of ssGSEA also indicated that VEGF-mediated ATOH8 upregulation may primarily depend on the AKT signalling pathway (Fig. 5g, Additional file 5: Table S3). As illustrated, blockade of AKT signalling partially reversed VEGF-induced upregulation of HK2 activity, and the expression of ATOH8, HK2, and BCL2/BAX, but restored the reduced ROS production caused by VEGF in CRC cells (Fig. 5h, Additional file 3: Figure S8f-g). And AKT inhibition could also partially reversed the protective effects of VEGF on cell survival in CRC m-CTCs (Fig. 5i). What’s more, ATOH8 overexpression attenuated AKT inhibitor (AZD5363) induced downregulation of ATOH8, HK2, and BCL2/BAX ratio, and partly reversed cell death in CRC m-CTCs (Fig. 5j-k). These findings reveal that VEGF upregulates ATOH8 by selectively activating VEGFR2-AKT signalling, with major implications for understanding and targeting the mechanism underlying m-CTC survival (Fig. 6).

## Discussion

Tumour physical microenvironment, especially the fluid microenvironment, plays an indispensable role in tumour metastasis [7]. Along with the invasion-metastasis cascade, tumour cells experience interstitial flow (~ 0.1 dyn/cm^2^), blood (1–30 dyn/cm^2^)/lymphatic circulation (~ 0.64 dyn/cm^2^), and target organ-specific fluid microenvironments [7]. Studies have proved that interstitial flow could enhance the invasion and metastasis ability of tumour cells, more specially, regulate the direction of tumour cell migration [38]. Furthermore, the incidence of individual organ metastasis is partially determined by organ blood flow [39]. However, the scarcity of CTCs in circulation [4] and the constantly changing blood flow are two main obstacles for researches on CTCs and LSS. Few studies have indicated that LSS promotes metastatic potential and anoikis resistance in breast CTCs [40, 41], but the effects of LSS on CRC CTCs are still poorly understood. In this article, we focused on only the initial shedding of CRC CTCs into the blood circulation and intended to explore the mechanobiological mechanisms of LSS regulation of CTC survival.

For CTCs, mechanical sensing molecules are essential for the process of responding to LSS and can convert mechanical stimuli into biochemical signals [15]. ATOH8 is such a shear stress sensor molecule, and its tumour-promoting effect in CRC still lacks strong evidence. Here, we confirmed that ATOH8 is associated with colon cancer hematogenous metastasis and poor prognosis in patients. Additionally, we found that ATOH8 was upregulated in CRC m-CTCs in response to LSS in vitro and in vivo. A previous study suggested that LSS can strengthen the interactions between CTCs and various blood components such as platelets, immune cells, and cytokines, to protect CTCs against death [42]. Interestingly, the present study elucidated another intrinsic survival mechanism in CTCs; that is, ATOH8 is elevated by LSS, similar to YAP, inhibiting cell death pathway in CRC m-CTCs [43]. Collectively, LSS upregulates ATOH8 expression in CRC m-CTCs and these ATOH8-overexpressing m-CTCs with pro-survival potential may exert essential effects in CRC metastasis.

Over the past years, exploring the intrinsic mechanism of CTC resistance to death has attracted attention. Numerous studies have shown that CTCs undergoing EMT [44] or with stem cell-like properties [45] have survival priorities. Additionally, survivin (+) CTCs can escape immune killing via blocking natural killer cell cytotoxicity [46]. HER2 (+) CTCs tend to survive by activating the PI3K and MAPK signalling pathways [47]. Thus, it is essential to identify the mechanism underlying the resistance to death in ATOH8 (+) or ATOH8-overexpressing m-CTCs. As established, metabolism and cell survival are inextricably linked, and cancer cells can flexibly switch between different metabolic states to cope with adverse conditions such as metabolic stress, anoikis, and mechanical stress [24, 48, 49]. Our ssGSEA analysis revealed that ATOH8 was involved in the glycolysis pathway, and we confirmed that silencing ATOH8 could reduce the glycolysis phenotype in suspended CRC cells. In fact, aerobic glycolysis, a central hallmark of tumours, is essential for tumour cell growth and survival under oxidative stressors such as anoikis and chemotherapy damage [50]. Indeed, we found that ATOH8 overexpression could promote CRC m-CTCs migration, invasion, anoikis resistance, and more importantly, could rescue CRC m-CTCs from 2-DG-induced cell death. Hence, ATOH8-mediated glycolysis may be an important factor facilitating CTC survival.

HK2 is one of the key enzymes of glycolysis, participating in the regulation of cancer cell metabolism and death, and its overexpression is significantly positively correlated with CRC recurrence [51]. Particularly, our data demonstrated that HK2 was stably upregulated in ATOH8-overexpressing CRC cells. Furthermore, ChIP and luciferase assay further indicated that HK2 is a direct target of ATOH8. It is reported that HK2 can support cell survival via promoting glycolysis and then reducing overabundant ROS or forming HK2-VDAC complex and then inhibiting mitochondria-mediated apoptosis. As expected, down-regulated ROS level and up-regulated mitochondrial HK2 were found in ATOH8-overexpressing CRC cells, and ATOH8 overexpression reversed CRC m-CTC death induced by the HK2 inhibitor, 3-BrPA. In summary, our work supported the view that the LSS-ATOH8-HK2 pathway is involved in the regulation of CTC survival, and thus yielding clues into a potential therapeutic strategy for CRC metastasis.

Furthermore, to clarify the effect of LSS on CTC survival in more detail, we elucidated the mechanobiological mechanism of ATOH8-meditated response to LSS in CRC m-CTCs. In the past, scholars discovered that LSS promoted VEGF secretion and inhibited cell apoptosis in endothelial cells [52–54]. Moreover, increasing evidence has indicated that LSS mediates tumour metastasis directly by acting on cytokines and their receptors in tumour cells, such as VEGF, IL11, and IGF-2 [32, 53, 55]. These suggest that the VEGF signalling pathway may serve as the bridge between LSS and ATOH8, and we did confirm that LSS induced the VEGF-VEGFR2 pathway, which regulated the ATOH8-mediated survival of m-CTCs. Additionally, the AKT pathway, as a classical downstream of VEGFR2 relating to cell survival, were found to partially mediate the ATOH8 upregulation and subsequently m-CTCs survival induced by VEGF. Here, a VEGF-VEGFR2-AKT signal axis in CRC m-CTCs was presented, which contributes to the high expression of ATOH8 and ultimately promotes CTC survival in the complex fluid microenvironment.

## Conclusions

Collectively, we have discovered a novel mechanobiological mechanism of m-CTC survival under LSS and demonstrated that ATOH8 suppressed cell death in CRC m-CTC, the critical steps in CRC metastasis. Mechanismly, ATOH8 was upregulated in CRC m-CTCs via the VEGF-VEGFR2-AKT signalling pathways triggered by LSS, consequently mediating m-CTC survival by primarily promoting HK2 transcriptional activity, which is of great significance for the effective prediction of tumour metastasis or the search for new CTC therapeutic targets.

## Supplementary information

**Additional file 1.** Supplementary methods and materials. More detailed information about CTCs isolation, drugs, animal studies, shear stress experiments, glycolysis assays, live/dead cell vitality assay, anoikis assay, ChIP, dual-luciferase reporter assay, ELISA, qPCR, WB, immunofluorescence, IHC, cell invasion assay, wound healing assay, cell-cycle analysis and bioinformatics analysis was addressed.

**Additional file 2:** **Table S1.** The real time polymerase chain reaction primers.

**Additional file 3: Figure S1.** Construction of mimic circulating tumour cells and the effects of LSS on CTCs. **Figure S2.** Shear stress responsive molecule ATOH8 is associated with poor prognosis in colorectal cancer patients. **Figure S3.** Overexpression of ATOH8 facilitates colorectal tumour cells to form metastases. **Figure S4.** ATOH8 promotes the invasion, metastasis and anoikis resistance of colorectal cancer cells. **Figure S5.** ATOH8 is associated with glycolysis in colorectal cancer. **Figure S6.** ATOH8 inhibits intravascular death of circulating colorectal tumour cells by targeting HK2. **Figure S7.** VEGF is responsible for ATOH8 upregulation in colorectal tumour cells in suspension and under LSS. **Figure S8.** VEGF-VEGFR2-AKT signalling axis activates ATOH8 and its downstream glycolysis pathway.

**Additional file 4: Table S2.** Demographics and clinical characteristics of 141 cases of CRC patients.

**Additional file 5: Table S3.** The results of ssGESA conducted in GSE131418.

**Additional file 6: Table S4.** Genes correlated with increased ATOH8 expression in GSE131418.

**Additional file 7: Table S5.** List of cytokines and cytokine receptor genes in GSEA.

## Data Availability

All data generated or analysed during this study are included in this published article (and its supplementary information files).

## Abbreviations

2-DG: 2-Deoxy-D-glucose
2-NBDG: 2-(N-(7-Nitrobenz-2-oxa-1,3-diazol-4-yl)Amino)-2-Deoxyglucose
3-BrPA: 3-bromopyruvate
ANOVA: Analysis of variance
ANTs: Adjacent normal tissues
ATOH8: Atonal bHLH transcription factor 8
ATP: Adenosine triphosphate
BAX: BCL2 associated X protein
BCL2: B cell lymphoma 2
CD45: Lymphocyte common antigen
ChIP: Chromatin immunoprecipitation
CK8: Cytokeratin 8
CRC: Colorectal cancer
CTCs: Circulating tumour cells
E-box: Enhancer box
EBV: Epstein-Barr virus
EMT: Epithelial–mesenchymal transition
ERK: Extracellular signal-regulated kinase
GFP: Green fluorescent protein
GLUT1: Glucose transporter 1
HER2: Human epidermal growth factor receptor 2
HK2: Hexokinase 2
HP: Hypertension
IGF-2: Insulin-like growth factor 2
IHC: Immunohistochemical
IL11: Interleukin 11
LDHA: Lactate dehydrogenase A
LSS: Laminar shear stress
MCT1: Monocarboxylate transporter 1
m-CTCs: Mimic circulating tumour cells
MTT: 3-(4,5-dimethylthiazol-2-yl)2,5-diphenyl tetrazolium bromide
NHP: Non-hypertension
OS: Overall survival
p38 MAPK: p38 mitogen-activated protein kinases
PANX1: Pannexin 1
PFS: Progression-free survival
PI: Propidium iodide
PI3K/AKT: Phosphoinositide 3 kinase/protein kinase B
qPCR: Quantitative polymerase chain reaction
ROS: Reactive oxygen species
SEM: Standard Error
SPSS: Statistical product and service solutions
ssGSEA: Single sample gene set enrichment analysis
TCGA: The cancer genome atlas
TSS: Transcription start site
VDAC: Voltage-dependent anion channel
VEGF: Vascular endothelial growth factor
VEGFR2: Vascular endothelial growth factor receptor 2
WB: Western blotting
YAP: Yes-associated protein
