# Supplementary material for: Shear stress activates ATOH8 via autocrine VEGF promoting glycolysis dependent-survival of colorectal cancer cells in the circulation
Source: J Exp Clin Cancer Res. 2020 Jan 30;39:25. doi: 10.1186/s13046-020-1533-0 (PMC6993408; doi:10.1186/s13046-020-1533-0)
Supplement: Supplementary file 1 — Additional file 1. Supplementary methods and materials. More detailed information about CTCs isolation, drugs, animal studies, shear stress experiments, glycolysis assays, live/dead cell vitality assay, anoikis assay, ChIP, dual-luciferase reporter assay, ELISA, qPCR, WB, immunofluorescence, IHC, cell invasion assay, wound healing assay, cell-cycle analysis and bioinformatics analysis was addressed. [file 13046_2020_1533_MOESM1_ESM.pdf]

Supplementary Methods and Materials

Circulating tumour cell (CTC) isolation and classification

CTCs were isolated and classified by epithelial-mesenchymal transition (EMT) markers using the CanPatrol™ CTC enrichment technique as previously described [1]. Briefly, a filter-based equipment was used to isolate CTCs by size and then Tri-colour RNA in situ hybridization (RNA-ISH) was performed to classify the different phenotype of CTCs according to EMT biomarkers. Cells treated with protease K (Qiagen GmbH, Hilden, Germany) at 25˚C for 1 h, were hybridized with capture probes and amplification probes, followed by 4',6-diamidino-2-phenylindole staining (Sigma-Aldrich; Merck KGaA, Darmstadt, Germany). Specific fluorescent signals represented different phenotype of CTCs were observed and photographed with Olympus BX53 fluorescence microscope (Olympus Corporation, Tokyo, Japan). The sequence of *ATOH8* capture probes is TGACGAGTAGGAACTTTCCCAGGAATCCTGGTGGTTATTGCTTTGATCTCsG GAGGAGGCGCATATGAGTAGCACGGCACCAGGATGGCCAGTTTGGACAGG ACAGGATGTAGTTACAGGCGGCACTGTAGTCAAGGTCAGACACACTCsGGA GAAGCTGAG.

Drugs

The drugs used are listed as follows: 2-Deoxy-D-glucose (2-DG) (HY-13966) and SCH772984 (HY-50846) as EKR inhibitor were purchased from MCE

(Monmouth Junction, NJ, USA); 3-bromopyruvate (3-BrPA) (S5426), VEGFR2 inhibitor ZM323881 (S2896) and Apatinib (S5248), AKT inhibitor AZD5363 (S8019) and MK-2206 (S1078) were purchased from Selleck (Houston, TX, USA); Recombinant Human VEGF 165 (HZ-1038; Proteintech, Wuhan, China) and anti-VEGF antibody bevacizumab (Avastin, Switzerland) were added into culture medium as intended.

RNA interference, plasmid transfection, and lentivirus transduction

Colorectal cancer cell lines LoVo and SW480 were transfected with small interfering RNA (siRNA) for gene silencing of ATOH8. The three ATOH8 siRNA sequences used were as follows: si-h-ATOH8_001 (5'GGATCGCCTGTAACTACAT dTdT 3'), si-h-ATOH8_002 (5' TCGTCAATTTCACACGTAA dTdT 3') and si-h-ATOH8_003 (5' GGCTGACCTTGACTACAGT dTdT 3'). For overexpression, the full-length ATOH8 was identified and cloned to generate a Flag-ATOH8 construct. To establish stable ATOH8 overexpression cells, a lentiviral vector containing green fluorescent protein (GFP) and luciferase reporters (pLV-EF1a-hluc-P2A-mNeongreen-CMV-MCS-3Xflag) was constructed and used to infect LoVo and SW480 cells, which were then selected with puromycin.

Animal studies

All animal experiments performed in this study were approved by the Institutional Animal Care and Use Committee of Nanfang Hospital, Southern medical

university. Female BALB/c nude mice aged 4–6 weeks were used in this study.

In order to explore the shear stress sensitivity of ATOH8 in colorectal cancer cells, resuspended 1×10^6 and 5×10^5 wild type SW480 cells in 100μl PBS were injected into mouse subcutaneously and intravenously simultaneously. Four weeks after injection, the mice were sacrificed and examined by H&E and IHC staining.

To evaluate the potential ability of $ATOH8^{high}$ mimic circulating tumour cells (m-CTCs) to promote cell survival, nude mice were intravenously injected with 1×10^6 vector or ATOH8 overexpressing luciferase-labelled SW480 cells, tumour cell accumulation was monitored by bioluminescence imaging systems (FX PRO, Bruker, Germany) every hour. Meanwhile, 1×10^6 vector or ATOH8 overexpressing GFP-labelled SW480 were intravenously injected into nude mice, and blood (300μl /per mouse) were obtained 0h, 4h, and 8h after injections. Firstly, the red blood cells in blood samples were destroyed. After centrifugation, 5μl of propidium iodide (PI, KGA108-2, Nanking, China) fluorescent dye was added to incubate at 25˚C for 10 min, and then the ratio of CTCs and PI+CTC was detected by flow cytometry.

To further explore the function of ATOH8 on hematogenous metastasis, nude mice were injected with 1×10^6 vector or ATOH8 overexpressing GFP-labelled SW480 cells. Four weeks later, mice were sacrificed, and lung tissues were harvested for metastasis evaluation as described. Additionally，blood (300μl /per mouse) were

obtained 4week after injections， and the ratio of CTCs and PI+CTC was detected by flow cytometry.

Shear stress experiments

The microfluidic system fabricated by 7 tandem μ-slides I 0.4 (diameter: long 50mm, width 5mm, and high 0.4mm) (Ibidi, GmbH, Martinsried, Germany), and infusion bump was used to load different level of shear stress on colorectal cancer cells (LoVo and SW480). Suspended cells (1×10^6 cells/ml) were injected into sterile infusion bag and flowed through transfusion facility which connected with the inlet of the microfluidic system. The flow rate of suspension was controlled by infusion bump while 228ml/h, 456 ml/h and 912 ml/h corresponded to 5dyn/cm^2, 10dyn/cm^2 and 20dyn/cm^2, respectively. Finally, cells returned to infusion bag after shear stress loading. All tubing and microfluidic devices were washed by sterile water for 5min for all experiments prior to injection of cell suspension at specific flow rate for 15min， 30min，60 min，2h and 4h. Cells were collected via the microfluidic system and used to conduct subsequent experiments.

Lactate production assay

Colorectal cancer cells (LoVo and SW480) were plated in 6-well plates and cultured until cell density reached to 30-40% followed by transfection with plasmid or siRNA of ATOH8 using Lipofectamine 2000 (11668030; Invitrogen, CA, USA). Then, cells were digested and seeded into 6-well low attachment plates for suspension

culture and subsequent treatment with cytokines or inhibitors for 24h. The detection of metabolite, ROS, enzyme activities and 2-NBDG uptake were performed. Cell supernatant was collected to measure lactate concentration using lactate detection kit (A019-2-1; Nanjing Jiancheng Bioengineering Institute, Nanjing, China) according to the manufacturer’ s protocol. The absorbance was measured at 530nm by a SpectraMax M5 microplate reader (Molecular Devices, Sunnyvale, CA).

HK2 activity

Cells were collected and ultrasonically schizolysised with 200μL lysis buffer prior to centrifugation at 8×10^3g for 10min at 4°C. 10μL of each supernatant was mixed with 180μL Glucose 6-phosphate and 10μL glucose 6-phosphate dehydrogenase (HK-1-Y; Comin, Suzhou, China). The absorbance was measured at 340nm using a SpectraMax M5 microplate reader (Molecular Devices, Sunnyvale, CA) at 20s and 5min20s, respectively. The protein level of each sample was also detected for standardization using BCA assay (P0011; Beyotime, Shanghai, China).

ROS measurement

Cells were cultured with medium containing fluorescent 2′, 7′-dichlorofluorescin diacetate (DCF-DA) for 30min to measure ROS level as described by manufacturer's instruction of commercial kit (E004-1-1; Nanjing Jiancheng Bioengineering Institute, Nanjing, China). Fluorescence quantification was conducted by flow cytometry at emission 525/excitation 485nm.

Live/dead cell vitality assay

To quantify live and dead cells, cells were collected and stained with Calcein-AM/ Propidium Iodide (PI) after shear stress (BB-4126-2; BestBio biotechnologies, Shanghai, China). Briefly, cells were washed with PBS and incubated in mixture containing 2μM Calcein-AM and 1μM PI for 15 min at 4℃ in the dark, followed by photographed (Calcein-AM: emission 490/excitation 515nm; PI: emission 488-545/excitation 617nm) with a fluorescence microscope (Olympus). And six representative images from different fields were used for subsequent statistical analysis.

Chromatin immunoprecipitation (ChIP)

ChIP assays were performed using a SimpleChIP Enzymatic Chromatin IP kit (no. 9003; Cell Signaling Technologies). LoVo and SW480 cells were transfected with a Flag-ATOH8 construct for 48h and then cross-linked with 37% formaldehyde at a final concentration of 1% at room temperature for 10 min. The fragmented chromatin was treated with nuclease and sonicated. Chromatin immunoprecipitation was performed with mouse anti DDDDK-Tag (AE005, ABclonal, Boston, USA), rabbit anti-histone H3, and normal rabbit IgG (a negative control). After reverse cross-linking and DNA purification, immunoprecipitated DNA was measured with gel electrophoresis with primers for predicted ATOH8 binding sites in the HK2 promoter (Additional file 2: Table S1).

Dual-Luciferase Reporter Assay

The HK2 vectors, including pGL4.10-HK2 wide type (wt) and pGL4.10-HK2 mutant types (mut1, mut2 and mut1+2), were constructed according to the gene symbol (HK2 NM_000189 human) and predicted binding sites. Cells were seeded with a concentration of 1.5×10^6 per well in 6-well plates followed by transfection with HK2 vectors, overexpressed-ATOH8 plasmid or control vector and plasmids renilla luciferase plasmid using Lipofectamine 2000. 36h later, the cells were collected to assess luciferase activity by a dual-luciferase reporter assay system (E1910; Promega, Madison, USA).

Measurement of VEGF levels in serum

Colorectal cancer cells (LoVo and SW480) were plated in 6-well plates and cultured until cell density reached to 30-40%. After transfected with plasmid or siRNA of ATOH8, cell supernatant was collected to quantify the cytokines VEGF by using their corresponding ELISA kits (E01V0016; MLBio, Shanghai, China).

Cellular ATP levels

Cell supernatant was harvested for ATP detection using a firefly luciferase-based ATP assay kit (S0027; Beyotime, Shanghai, China) as previously described [2]. In brief, 20μL of each supernatant together with 10μL ATP detection working dilution was used to measure luminance (RLU) value using a SpectraMax M5 microplate

reader (Molecular Devices, Sunnyvale, CA). The protein levels were detected using BCA assay and cellular relative ATP levels were calculated by the ratio of ATP value to protein value.

2-NBDG uptake assay

Cells were cultured with glucose-free medium for 12h and then incubated with 100μM 2-NBDG (N13195; Life Technologies, Eugene, Oregon, OR, USA) for 30min at 37°C. Fluorescence quantification was analysed by flow cytometry at emission 465/excitation 540nm.

Anoikis assay

Cells were seeded in 24-well low attachment plates or control plates for anoikis detection using CytoSelect 24-Well Anoikis Assay Kit (CBA-080; Cell Biolabs, Inc., San Diego, CA) [3]. Briefly, cells were incubated with calcein AM/ EthD-1 solution (500X, 1 μl) for 60min at 37°Cprior to fluorescence photography or MTT assay.

Quantitative Real-Time Polymerase Chain Reaction (qRT-PCR)

Total RNA was extracted using Trizol kit according to the manufacturer's protocol and reverse transcribed with Takara RT reagent (RR037B; Takara Bio, Shiga, Japan). qRT-PCR was conducted using LightCycler 480 system Version 1.5 (Roche, Penzberg, Germany) as described previously [2]. The primer sequences used for qRT-PCR are listed in (Additional file 2: Table S1).

Western Blotting

Western blotting was performed as previously described [4]. Immunoblots were detected with fluorophore-conjugated goat anti-rabbit or anti-mouse secondary antibodies (1:1000, LI-COR, Lincoln, NE, USA) by an Odyssey imaging system (LI-COR). Antibodies used were as followed: anti-ATOH8 (1:1000, DF10047; Affinity Biosciences, OH, USA), anti-HK2 (1:1000, 22029-1-AP; Proteintech, Wuhan, China), anti-VEGF (1:750, WL00009b; Wanlei Life Science, Shenyang, China), anti-VEGFR2 (1:1000, WL02294; Wanlei Life Science, Shenyang, China), anti-VDAC (1:1000, WL02790; Wanlei Life Science, Shenyang, China), anti-BAX (1:1500, ab182733; Abcam, Cambridge, MA, USA), anti-BCL2 (1:1000, 12789-1-AP; Proteintech, Wuhan, China), anti-GLUT1 (1:1000, bs-4855R; Bioss Biotechnology Company, Beijing, China) , anti-LDHA (1:1000, bs-1810R; Bioss Biotechnology Company, Beijing, China) , anti-AKT (1:1000, 4691; Cell Signaling Technology, Danvers, MA, USA), anti-p-AKT (Ser473) (1:1000, 4060; Cell Signaling Technology, Danvers, MA, USA), anti-ERK (1:1000, 4695; Cell Signaling Technology, Danvers, MA, USA), anti-p-ERK (1:1000, Tyr-202/ Tyr-204, 4370; Cell Signaling Technology, Danvers, MA, USA) anti-ATP5A1 (1:500, bs-2435R; Bioss Biotechnology Company, Beijing, China), anti-GAPDH (1:5000, bs-0755R; Bioss Biotechnology Company, Beijing, China) and anti-β-actin(1:5000, bsm-33036M; Bioss Biotechnology Company, Beijing, China)

Immunofluorescence (IF) staining and Immunohistochemistry (IHC) staining

IF and IHC was performed as previously described [3]. Briefly, the cells were incubated with the ATOH8 antibody (1:250, DF10047; Affinity Biosciences, OH, USA), CD45 antibody (1:200, 14-0451-81; Invitrogen, CA, USA), CK8 antibody (1:200, WL02755; Wanlei Life Science, Shenyang, China), overnight at 4°C, and the secondary antibody Alexa Fluor 555-labeled Donkey Anti-Mouse IgG (1:200, A0460; Beyotime, Shanghai, China) and Alexa Fluor 488-labeled Goat Anti-Rabbit IgG (1:200, A0423; Beyotime, Shanghai, China) were used for 1 hour. Then, DAPI (1:1000, C1002; Beyotime, Shanghai, China) diluted with methanol and incubated at room temperature for 10 min. Additionally, photos were taken using fluorescence and laser confocal microscopes. IHC staining was applied to detect the expression levels of ATOH8 (1:200), HK2 (1:200), GLUT1 (1:200), LDHA (1:200), Cleaved caspase 3 (1:150, WL01992; Wanlei Life Science, Shenyang, China), and Ki67 (1:100, ab15580; Abcam, Cambridge, MA, USA). Moreover, the expressions of ATOH8, HK2, GLUT1, LDHA, Ki67 and cleaved caspase 3 were quantified by using ImageJ analysis with novel immunohistochemistry profiler plugin [5].

MTT assay

Cells were seeded into 96-well plates at a concentration of 1×10^3 cells per well and transfected with plasmid using Lipofectamine 2000. After 48h, thiazolyl blue (3-(4,5-dimethyl-2-thiazolyl)-2,5-diphenyltetrazolium bromide, MTT) was added to the cells and incubated for 4-6h at 37°C, followed by replacement of 150μl per well

dimethyl sulfoxide (DMSO) as previously reported. Absorbance was measured at 570nm using a SpectraMax M5 microplate reader (Molecular Devices, Sunnyvale, CA).

Cell invasion assay

The upper chamber of 24-well transwell was added 100μl 1mg/mL matrigel diluted with serum free-cold media and incubated at 37°C for gelling. About 5h later, 200μl cell suspension with serum free media was put onto the matrigel, while the lower chamber of the transwell was filled with 500μl media containing 10% FBS. After incubation at 37°C for 48h, cells invaded through matrigel to the lower surface were fixed with 4% paraformaldehyde and stained with hematoxylin. The invaded cells were quantified and photographed under microscope.

Wound healing assay

Cells were plated in 6-well plates at a concentration of 5×10^5 cells per well and cultured until confluent. A 200μl pipette tip was used to make a straight scratch, simulating a wound. Wound closure was observed at 0, 24 and 48h under an inverted microscope (Olympus Corporation, Tokyo, Japan).

Cell-cycle analysis

The cell cycle was analysed using cell cycle staining kit (MultiSciences, Hangzhou, China). Briefly, Cells were harvested and incubated with mixture

containing 1ml DNA staining solution and 10μl permeabilization solution for 30min at room temperature in the absence of light. The cell-cycle phases were analysed by flow cytometry system (Beckman Coulter, Indianapolis, IN) at an excitation wavelength of 488 nm and an emission wavelength of 525 nm.

Bioinformatics analysis

Gene expression data and clinical data of 208 CRC patient samples were downloaded from TCGA (https://cancergenome.nih.gov/) on November 8, 2017, the GSE131418, GSE103479, GSE13712 and GSE52211 data were downloaded from GEO (https://www.ncbi.nlm.nih.gov/gds/). The list of gene sets such as cytokine and receptor genes was downloaded from GSEA (http://software.broadinstitute.org/gsea/index.jsp). Kaplan–Meier analysis of OS and PFS corresponding to the expression of *ATOH8* was analysed by GraphPad. Single sample Gene Set Enrichment analysis (ssGSEA) methods were available from the GSVA package [6]. R package limma was employed to perform gene expression analyse in R 3.6.0. Fold change > 1.5 and adjusted P value <0.05 were set as the threshold for significant differential expression.

References

1. Li Y, Huang N, Wang C, Ma H, Zhou M, Lin L, et al. Impact of liver tumor percutaneous radiofrequency ablation on circulating tumor cells. Oncology Letters. 2018;16:2839-50.

2. He W, Liang B, Wang C, Li S, Zhao Y, Huang Q, et al. MSC-regulated lncRNA MACC1-AS1 promotes stemness and chemoresistance through fatty acid oxidation in gastric cancer. ONCOGENE. 2019;38:4637-54.

3. Tan Y, Lin K, Zhao Y, Wu Q, Chen D, Wang J, et al. Adipocytes fuel gastric cancer omental metastasis via PITPNC1-mediated fatty acid metabolic reprogramming. Theranostics. 2018;8:5452-68.

4. Hao S, Yu J, He W, Huang Q, Zhao Y, Liang B, et al. Cysteine Dioxygenase 1 Mediates Erastin-Induced Ferroptosis in Human Gastric Cancer Cells. NEOPLASIA. 2017;19:1022-32.

5. Mane DR, Kale AD, Belaldavar C. Validation of immunoexpression of tenascin-C in oral precancerous and cancerous tissues using ImageJ analysis with novel immunohistochemistry profiler plugin: An immunohistochemical quantitative analysis. J Oral Maxillofac Pathol. 2017;21:211-7.

6. Shen S, Wang G, Zhang R, Zhao Y, Yu H, Wei Y, Chen F. Development and validation of an immune gene-set based Prognostic signature in ovarian cancer. EBioMedicine. 2019;40:318-26.
