# Supplementary material for: Shear stress activates ATOH8 via autocrine VEGF promoting glycolysis dependent-survival of colorectal cancer cells in the circulation
Source: J Exp Clin Cancer Res. 2020 Jan 30;39:25. doi: 10.1186/s13046-020-1533-0 (PMC6993408; doi:10.1186/s13046-020-1533-0)
Supplement: Supplementary file 2 — Additional file 2: Table S1. The real time polymerase chain reaction primers. [file 13046_2020_1533_MOESM2_ESM.pdf]

Table S1 Real-Time Polymerase Chain Reaction Primers

Name		Sequence
ATOH8	Forward (5’-3’)	CAGGTGCCGTGCTACTCATA
	Reverse (5’-3’)	AGTCACTCCTTGCGCTTCTT
GAPDH	Forward (5’-3’)	ACCCAGAAGACTGTGGATGG
	Reverse (5’-3’)	TCTAGACGGCAGGTCAGGTC
HK2	Forward (5’-3’)	GAGCCACCACTCACCCTACT
	Reverse (5’-3’)	CCAGGCATTCGGCAATGTG
BAX	Forward (5’-3’)	CCCGAGAGGTCTTTTTCCGAG
	Reverse (5’-3’)	CCAGCCCATGATGGTTCTGAT
BCL2	Forward (5’-3’)	GGTGGGGTCATGTGTGTGG
	Reverse (5’-3’)	CGGTTCAGGTACTCsAGTCATCC
MMP9	Forward (5’-3’)	GGGACGCAGACATCGTCATC
	Reverse (5’-3’)	TCGTCATCGTCGAAATGGGC
MMP1	Forward (5’-3’)	GGGGCTTTGATGTACCCTAGC
	Reverse (5’-3’)	TGTCACACGCTTTTGGGGTTT
CD80	Forward (5’-3’)	AAACTCGCATCTACTGGCAAA
	Reverse (5’-3’)	GGTTCTTGTACTCGGGCCATA
TIMP1	Forward (5’-3’)	CTTCTGCAATTCCGACCTCGT
	Reverse (5’-3’)	ACGCTGGTATAAGGTGGTCTG
IL11	Forward (5’-3’)	CGAGCGGACCTACTGTCCTA
	Reverse (5’-3’)	GCCCAGTCAAGTGTCAGGTG
IL10	Forward (5’-3’)	GATGTCAAACTCACTCATGGCT
	Reverse (5’-3’)	CGAGCGGACCTACTGTCCTA
IFNG	Forward (5’-3’)	TCGGTAACTGACTTGAATGTCCA
	Reverse (5’-3’)	TCGCTTCCCTGTTTTAGCTGC
ANG	Forward (5’-3’)	CTGGGCGTTTTGTTGTTGGTC
	Reverse (5’-3’)	GGTTTGGCATCATAGTGCTGG
TGF-β3	Forward (5’-3’)	ACAACACCTGAACCCAGAG

	Reverse (5’-3’)	ACTGCAGTGAGCAAGCTGA
TNF	Forward (5’-3’)	AAGCCTGTAGCCCATGTTGTAGC
	Reverse (5’-3’)	GAAGACCCCTCCCAGATAGATG
VEGFR2	Forward (5’-3’)	GGCCCAATAATCAGAGTGGCA
	Reverse (5’-3’)	CCAGTGTCATTTCCGATCACTTT
GLUT1	Forward (5’-3’)	GCCAGAAGGAGTCAGGTTCAA
	Reverse (5’-3’)	TCCTCGGAAAGGAGTTAGATCC
LDHA	Forward (5’-3’)	ATGGCAACTCTAAAGGATCAGC
	Reverse (5’-3’)	CCAACCCCAACAACTGTAATCT
VEGF	Forward (5’-3’)	AGGGCAGAATCATCACGAAGT
	Reverse (5’-3’)	AGGGTCTCGATTGGATGGCA
E-Cadherin	Forward (5’-3’)	CGAGAGCTACACGTTCACGG
	Reverse (5’-3’)	GGGTGTCGAGGGAAAAATAGG
N-Cadherin	Forward (5’-3’)	TGCGGTACAGTGTAACTGGG
	Reverse (5’-3’)	GAAACCGGGCTATCTGCTCG
Vimentin	Forward (5’-3’)	GACGCCATCAACACCGAGTT
	Reverse (5’-3’)	CTTTGTCGTTGGTTAGCTGGT
Laminin5	Forward (5’-3’)	GACTGCCAACAGTGCCAAC
	Reverse (5’-3’)	CCACCCTGATAGGTGCCAT
Fibronectin	Forward (5’-3’)	CGGTGGCTGTCAGTCAAAG
	Reverse (5’-3’)	AAACCTCGGCTTCCTCCATAA
HK2-p1	Forward (5’-3’)	TAAGTGGTCAGATGCACGGT
	Reverse (5’-3’)	CCCTCACAGCCAGTCAATCC
HK2-p2	Forward (5’-3’)	GTTGAGTTCAGCTTTGGACATGTT
	Reverse (5’-3’)	AGAAACTAACTTCCTAGCCCCATG
HK2-p3	Forward (5’-3’)	GAGCTCAATTCTGTGTGGAGT
	Reverse (5’-3’)	ACTTCTTGAGAACTATGTACCCTT
