# Supplementary material for: Shear stress activates ATOH8 via autocrine VEGF promoting glycolysis dependent-survival of colorectal cancer cells in the circulation
Source: J Exp Clin Cancer Res. 2020 Jan 30;39:25. doi: 10.1186/s13046-020-1533-0 (PMC6993408; doi:10.1186/s13046-020-1533-0)
Supplement: Supplementary file 3 — Additional file 3: Figure S1. Construction of mimic circulating tumour cells and the effects of LSS on CTCs. Figure S2. Shear stress responsive molecule ATOH8 is associated with poor prognosis in colorectal cancer patients. Figure S3. Overexpression of ATOH8 facilitates colorectal tumour cells to form metastases. Figure S4. ATOH8 promotes the invasion, metastasis and anoikis resistance of colorectal cancer cells. Figure S5. ATOH8 is associated with glycolysis in colorectal cancer. Figure S6. ATOH8 inhibits intravascular death of circulating colorectal tumour cells by targeting HK2. Figure S7. VEGF is responsible for ATOH8 upregulation in colorectal tumour cells in suspension and under LSS. Figure S8. VEGF-VEGFR2-AKT signalling axis activates ATOH8 and its downstream glycolysis pathway. [file 13046_2020_1533_MOESM3_ESM.pdf]

Supplementary figures

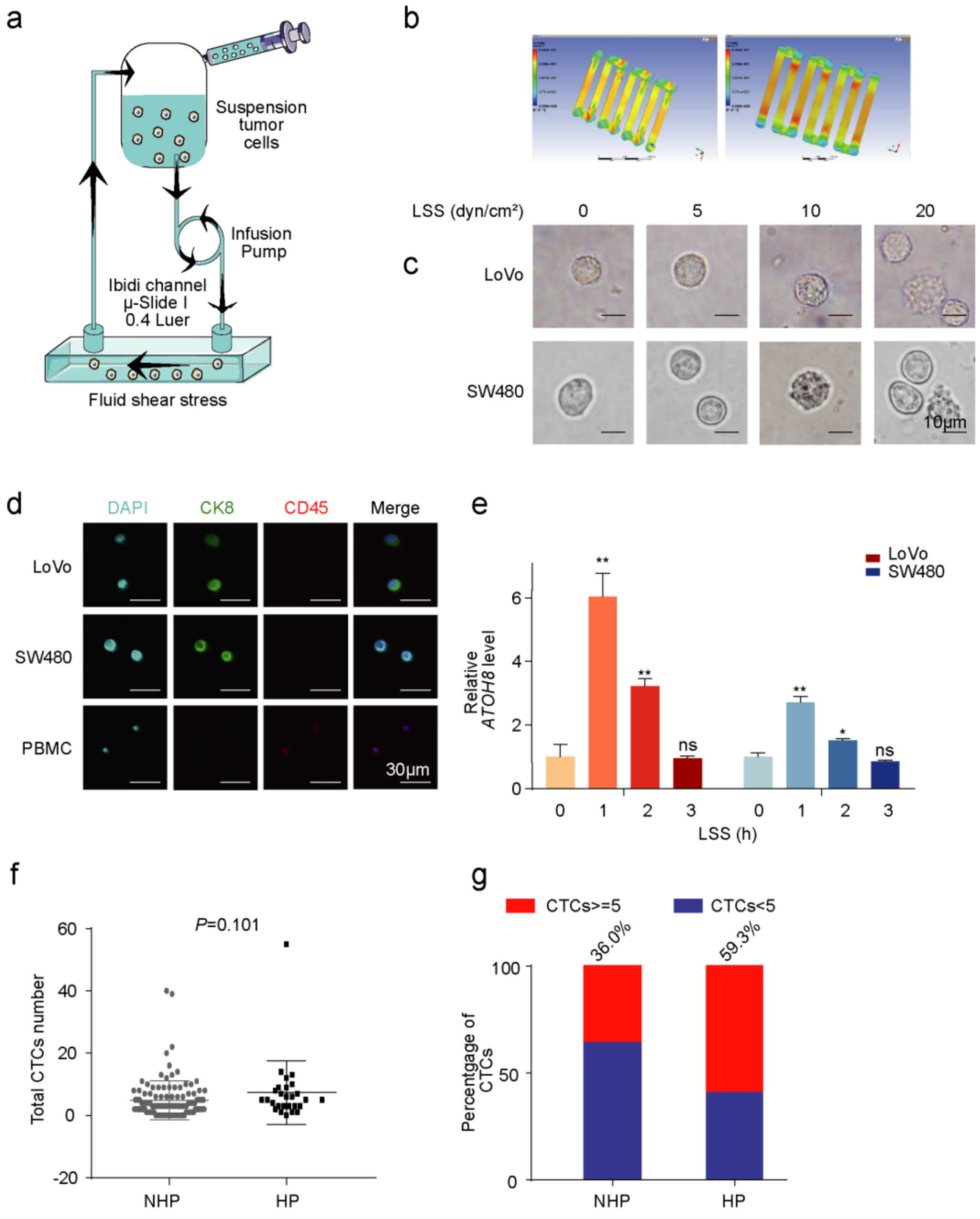

Figure S1. Construction of mimic circulating tumour cells and the effects of LSS on CTCs. a Schematic diagram of laminar shear stress (LSS) loading platform *in vitro*. **b** Flow velocity map simulated by ANSYS software, to verify that this LSS loading system consisting of seven IBIDI channels in series can generate a relatively stable and uniform LSS. **c** The suspended LoVo and SW480 cells can maintain normal

cell morphology after 30 min of shear stress stimulation in the physiological range of 0-20 dyn/cm^2. **d** Representative immunofluorescence images of DAPI, CD45, and CK8 expression in suspended LoVo and SW480 cells and peripheral blood mononuclear cells (PBMCs). **e** Quantitative polymerase chain reaction (qPCR) analysis of ATOH8 expression in suspended LoVo and SW480 cells treated with longer time gradient (10 dyn/cm^2; 0, 1, 2, 4h) LSS. **f** In colorectal cancer patients with or without hypertension, the total number of CTCs detected. **g** The percentage of colorectal cancer patients with CTCs ≥ 5 were higher in hypertension group. *$P < 0.05$ and **$P < 0.01$.

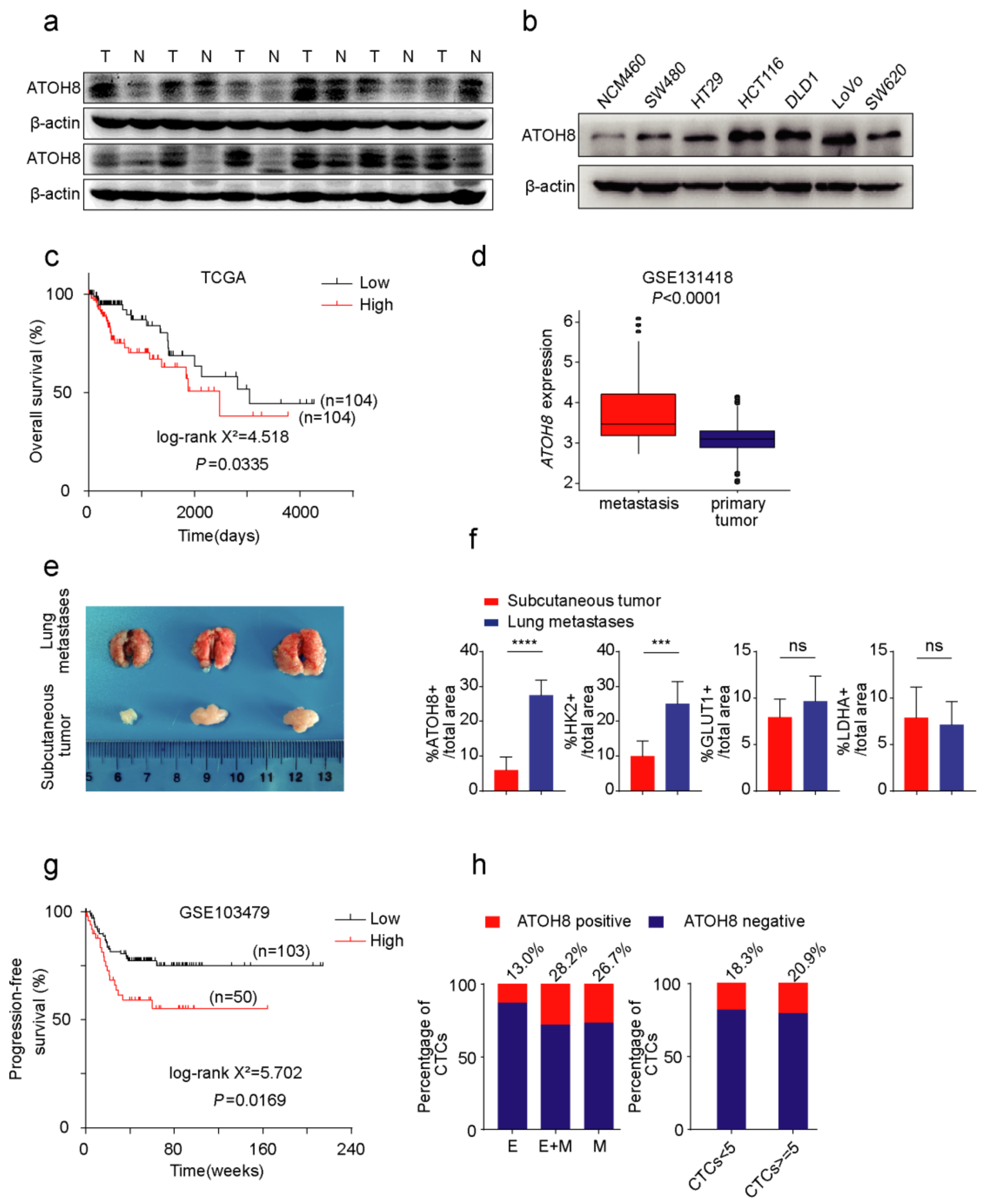

Figure S2. Shear stress responsive molecule ATOH8 is associated with poor prognosis in colorectal cancer patients. a Western blot (WB) analysis showed the ATOH8 expression from 12 colorectal cancer patients with tumour tissues and matched adjacent normal tissues. **b** WB results showing baseline protein levels of ATOH8 in NCM460 and six colon cancer cell lines. **c** Kaplan-Meier analysis of overall survival according to the expression of the ATOH8 in 208 colorectal cancer

patients from TCGA. **d** The expression of *ATOH8* in colorectal cancer metastases was significantly higher than that in primary tumours in GSE131418. **e** Gross view of lung metastasis and subcutaneous tumour from nude mice. **f** ATOH8, HK2, GLUT1 and LDHA protein expression quantified via immunohistochemically staining intensity based on Fig 1i. **g** Kaplan-Meier analysis of progression-free survival according to the expression of the ATOH8 in 153 surgically treated patients with stage II-III colorectal cancer from GSE103479. **h** The proportion of ATOH8-positive CTCs from 141 colorectal cancer patients were detected and quantified in different groups.

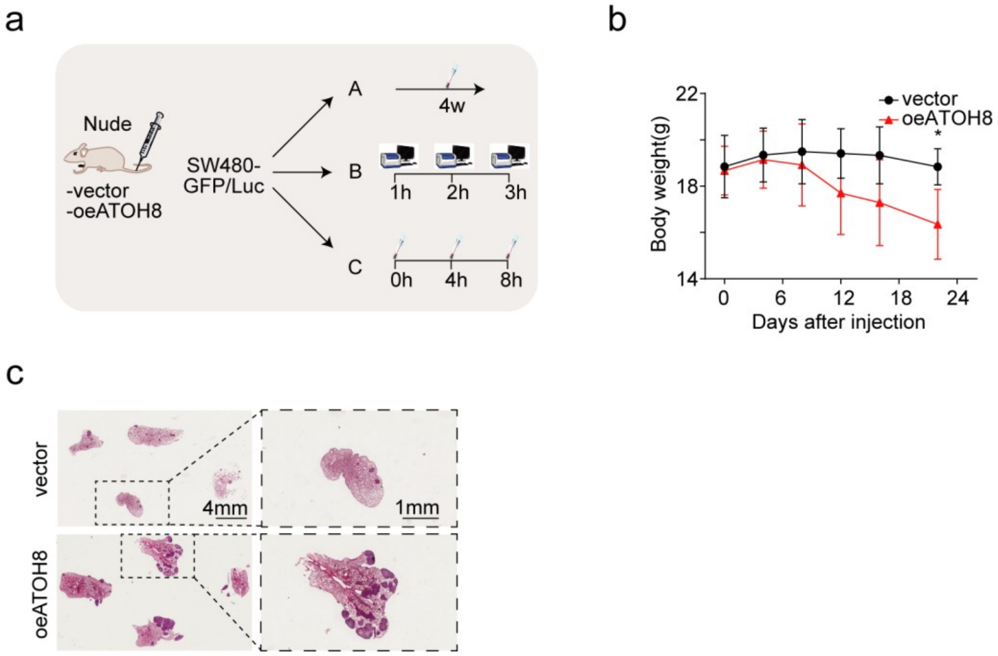

Figure S3. Overexpression of ATOH8 facilitates colorectal tumour cells to form metastases. a Experimental scheme detailing observation times and detection indicators of *in vivo* experiment. **b** Vector or ATOH8-overexpressing SW480 cells with GFP labelling were injected intravenously into nude mouse, and the mice body weight was tested within 3 weeks. **c** Representative images of haematoxylin and eosin staining of metastatic nodules in the lungs from vector or ATOH8-overexpressing group. *$P < 0.05$.

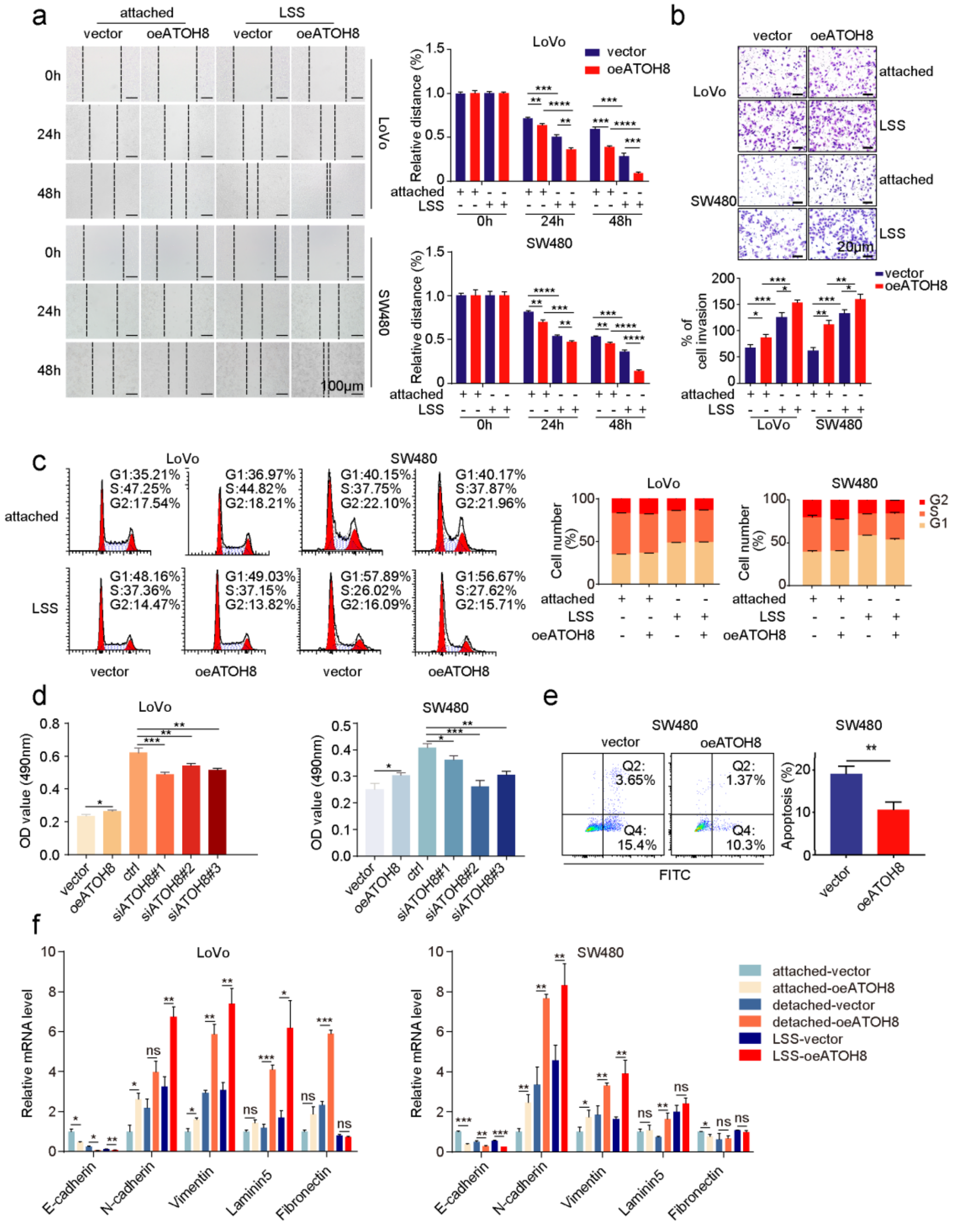

Figure S4. ATOH8 promotes the invasion, metastasis and anoikis resistance of colorectal cancer cells. a Wound healing assay was performed to compare cell migration ability between vector or ATOH8-overexpressing LoVo and SW480 cells with or without LSS (10 dyn/cm^2, 30min) stimulation. The representative plots (Left)

and quantification (Right) results were presented. **b** Matrigel invasion assay was performed to compare cell invasion ability between vector or ATOH8-overexpressing LoVo and SW480 cells with or without LSS (10 dyn/cm^2, 30min) stimulation. The representative plots (Upper) and quantification (Down) results were presented. **c** Flow cytometry analysis of cell cycle phase distribution of vector or ATOH8-overexpressing LoVo and SW480 cells with or without LSS (10 dyn/cm^2, 30min) stimulation. The representative plots (Left) and quantification (Right) results were presented. **d** MTT assays were conducted to measure the anoikis rate of suspended LoVo and SW480 cells after overexpressing or silencing ATOH8. **e** Apoptosis rates of suspended SW480 cells with or without ATOH8 overexpression were measured using flow cytometry with double staining of Annexin V and PI. Left, representative scatter plots of PI vs. Annexin V, while percentage of apoptotic cells was shown in Right. **f** qPCR analysis of the expression level of anoikis markers, including *E-acdherin*, *N-cadherin*, *Vimentin*, *Laminin5* and *Fibronecctin* in vector or ATOH8-overexpressing LoVo and SW480 cells with attached, detached or LSS (10 dyn/cm^2, 30min) stimulation. *$P < 0.05$, **$P < 0.01$, ***$P < 0.001$ and ****$P < 0.0001$.

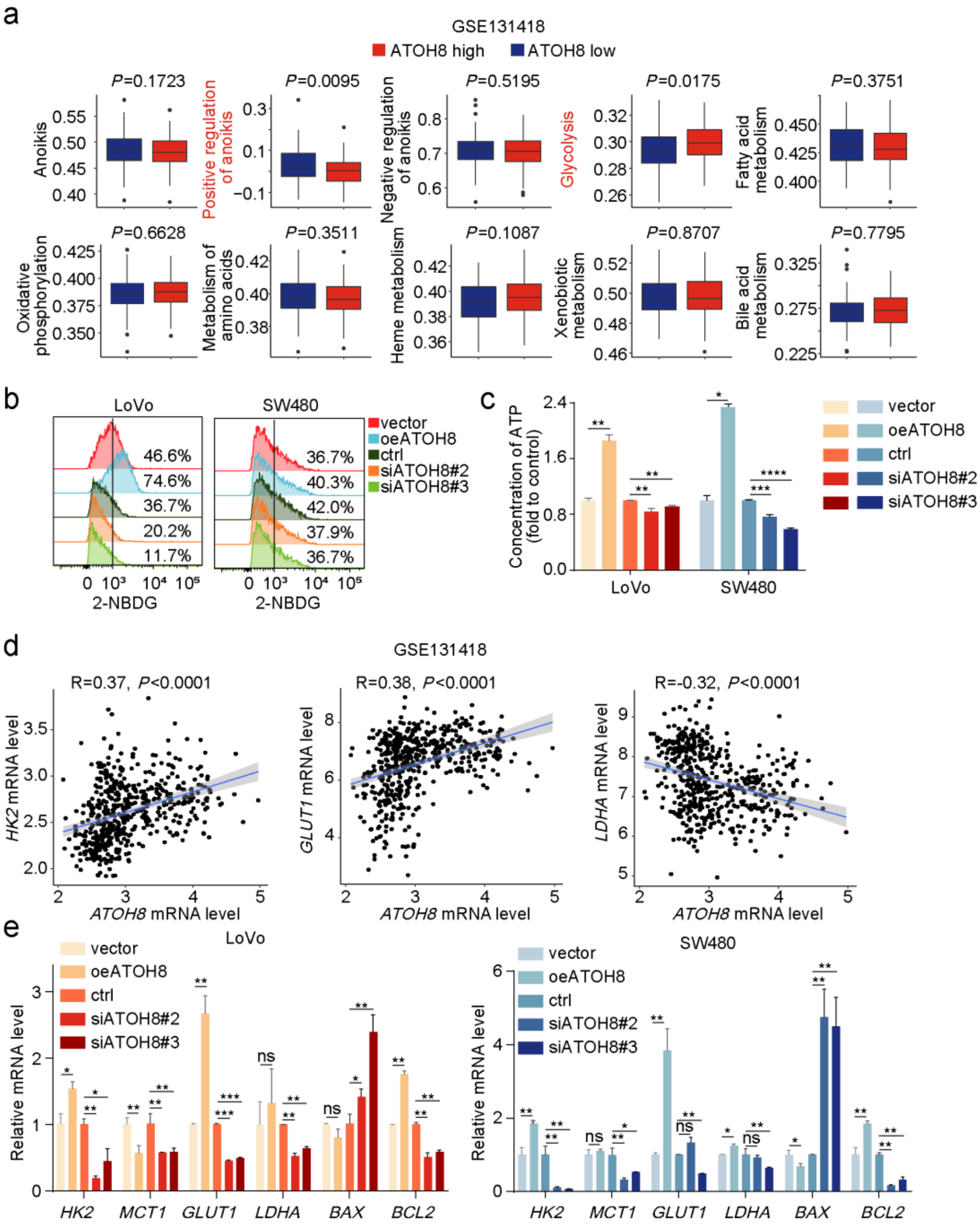

Figure S5. ATOH8 is associated with glycolysis in colorectal cancer. a Single sample gene set enrichment analysis (ssGSEA) of gene-containing signature in $ATOH8_{high}$ and $ATOH8_{low}$ group in the colorectal cancer metastasis cohort from GSE131418 and the results of anoikis-related and key metabolic pathways were presented. **b** 2-NBDG uptake assays showing that ATOH8 promoted glucose absorption in suspended LoVo and SW480 cells. **c** Overexpression of ATOH8

promoted ATP production in suspended LoVo and SW480 cells, while opposite effects were observed when silencing ATOH8. **d** Pearson correlation analysis of the expression of *ATOH8* and the glycolytic enzymes *HK2, LDHA* and *GLUT1* in colorectal cancer tissues from GSE131418. **e** qPCR analysis of the expression level of glycolytic enzymes *HK2*, *LDHA*, *GLUT1* and *MCT1* and apoptotic markers *BAX*, *BCL2* in LoVo and SW480 cells after overexpressing or silencing ATOH8. *$P < 0.05$, **$P < 0.01$, ***$P < 0.001$ and ****$P < 0.0001$.

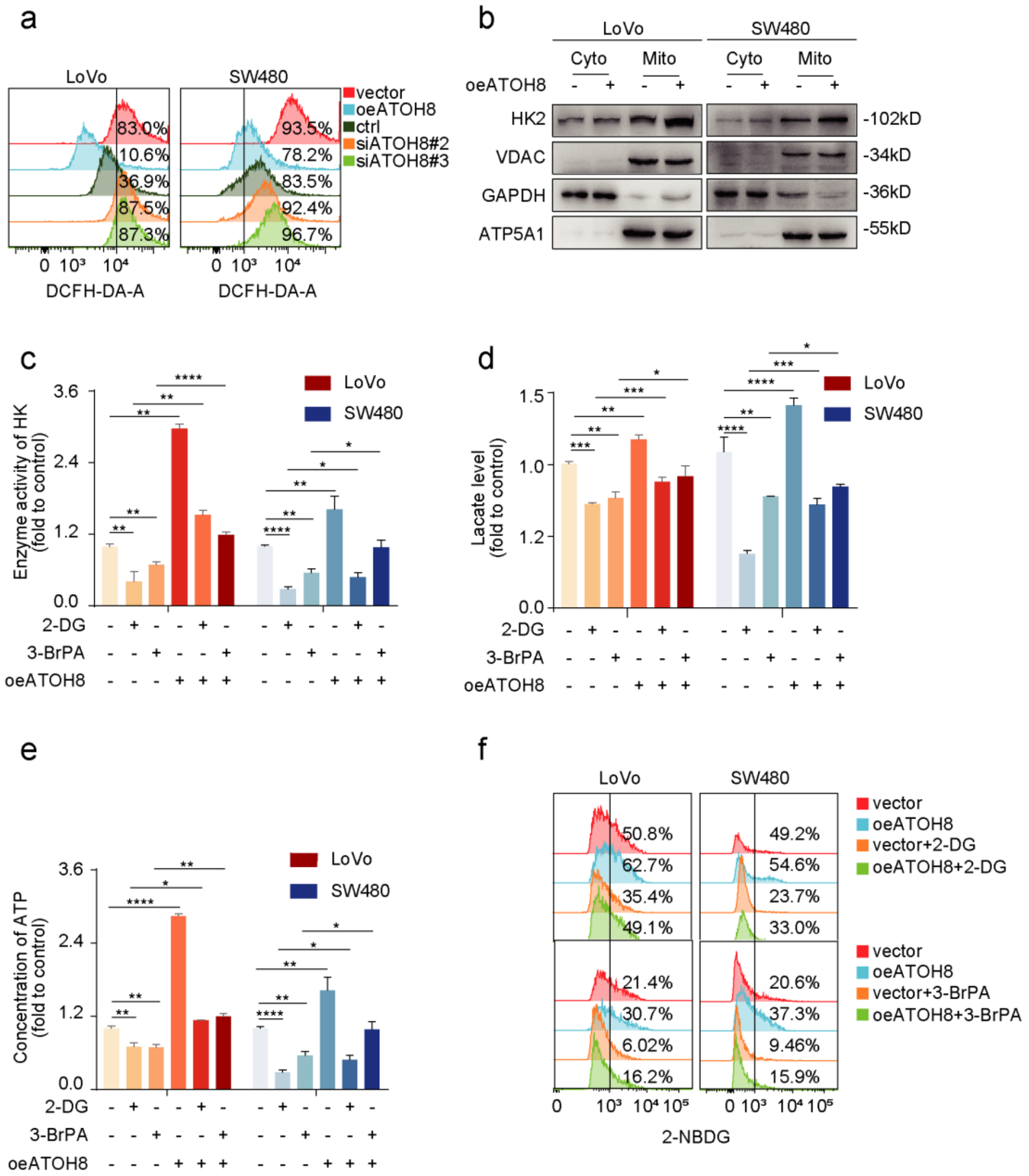

Figure S6. ATOH8 inhibits intravascular death of circulating colorectal tumour cells by targeting HK2. a ROS assay kit was used to measure ROS accumulation in LoVo and SW480 cells with ATOH8 overexpression or silencing. **b** WB results showing that ATOH8 promote the distribution of HK2 in the cytoplasm and especially mitochondria. **c-f** HK2 enzyme activity (**c**), lactate production (**d**), ATP (**e**), glucose absorption (**f**) in LoVo and SW480 cells after overexpressing ATOH8 and treating with or without 1 mM 2-Deoxy-D-glucose (2-DG) or 2 nM 3-bromopyruvate (3-BrPA)

for 24 h. *$P < 0.05$, **$P < 0.01$, ***$P < 0.001$ and ****$P < 0.0001$.

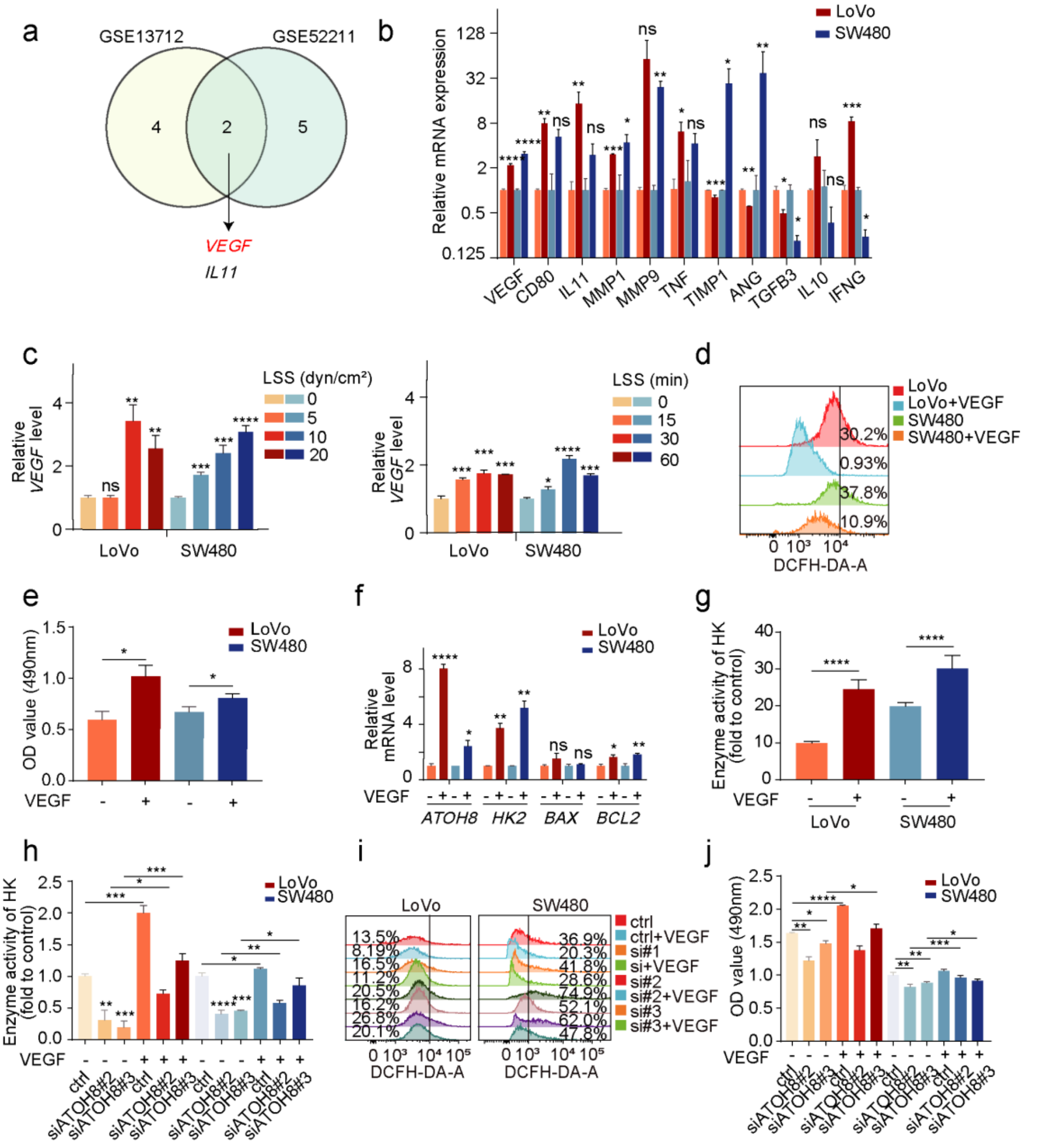

Figure S7. VEGF is responsible for ATOH8 upregulation in colorectal tumour cells in suspension and under LSS. a Venn diagram showing the number of differentially expressing cytokines and cytokine receptors in endothelial cells undergoing LSS in GSE13712 and GSE52211. **b** The qPCR analysis of 11 cytokines or cytokine receptors expression in suspended LoVo and SW480 cells treated with LSS (10dyn/cm^2, 30min). **c** The qPCR analysis of *VEGF* expression in suspended LoVo and SW480 cells treated with size gradient (0,5,10,20 dyn/cm^2; 30min; Left)

and time gradient (10 dyn/cm^2; 0,15, 30, 60min; Right) LSS. **d, e** ROS accumulation (**d**) and MTT assay (**e**) analysis in suspended LoVo and SW480 cells treated with 10ng/mL VEGF for 24h. **f, g** The qPCR analysis of expression level of *ATOH8*, *HK2*, *BAX* and *BCL2* (**f**) and HK2 enzyme activity (**g**) in suspended LoVo and SW480 cells treated with 10ng/mL VEGF for 24h. **h-j** Suspended LoVo and SW480 cells transfected with ctrl or si-ATOH8 were seeded in low attachment 6-well plate and treated with 10ng/mL VEGF for 24h. HK2 enzyme activity (**h**), ROS accumulation (**i**) and cell vitality (**j**) were measured. *$P < 0.05$, **$P < 0.01$, ***$P < 0.001$ and ****$P < 0.0001$.

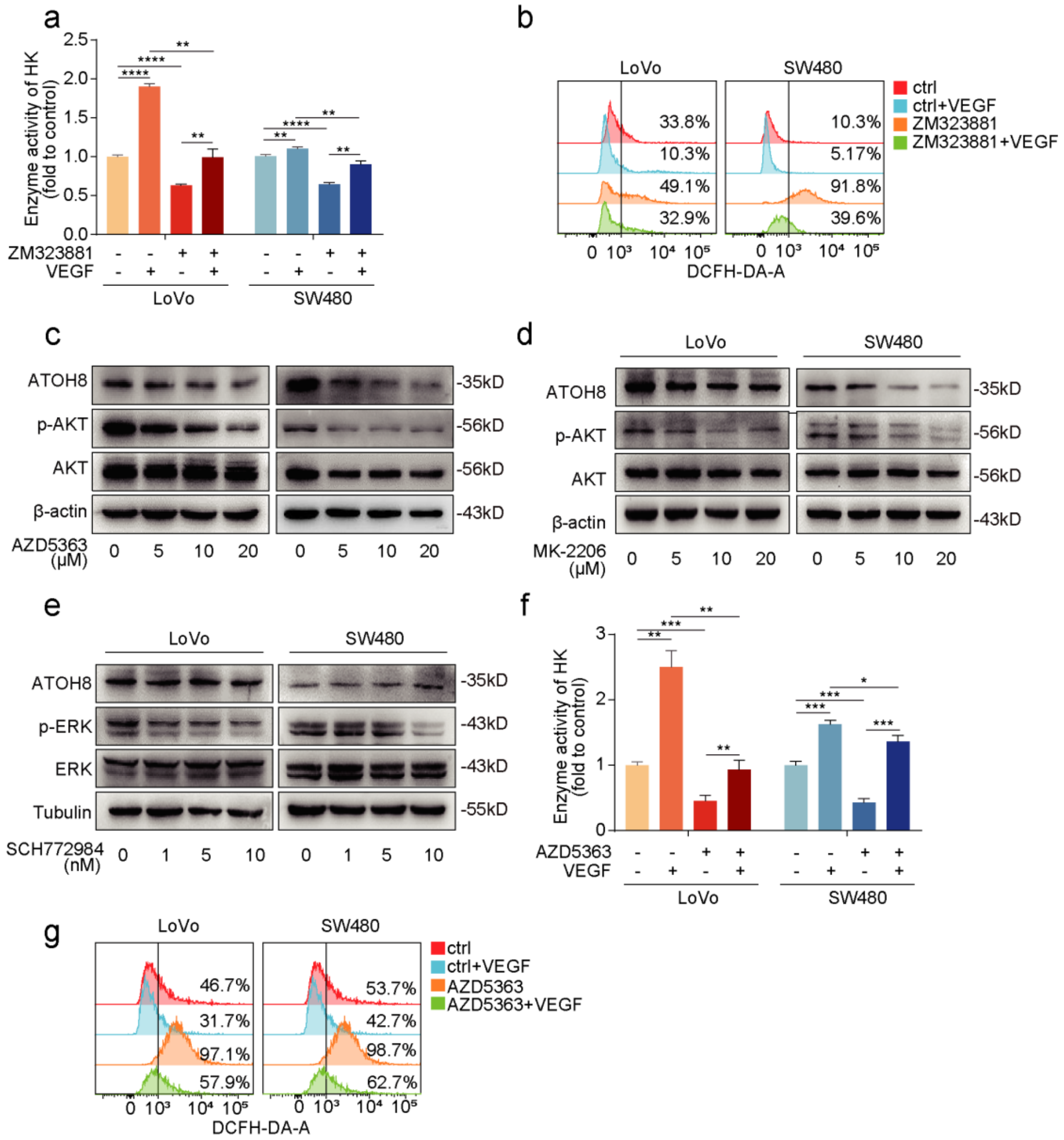

Figure S8. VEGF-VEGFR2-AKT signalling axis activates ATOH8 and its downstream glycolysis pathway. a, b Suspended LoVo and SW480 cells treated with 10ng/mL VEGF for 24h and with or without 10 μM VEGFR2 inhibitor (ZM323881), HK2 enzyme activity (**a**) and ROS accumulation (**b**) were analysed. **c, d** LoVo and SW480 cells was treated with AKT inhibitors AZD5363 (0, 5, 10, 20 μM) (**c**) or MK-2206 (0, 5, 10, 20 μM) (**d**), and the relative change in ATOH8, AKT, p-AKT expression was analysed by WB. **e** LoVo and SW480 cells was treated with ERK inhibitors SCH772984 (0, 1, 5, 10 nM), and the relative change in ATOH8, ERK,

p-ERK expression was analysed by WB. **f, g** Suspended LoVo and SW480 cells treated with 10ng/mL VEGF for 24h and with or without 10 μM AKT inhibitor (AZD5363), HK2 enzyme activity (**f**) and ROS accumulation (**g**) were analysed. *$P < 0.05$, **$P < 0.01$, ***$P < 0.001$ and ****$P < 0.0001$.
