# Supplementary material for: Shear stress activates ATOH8 via autocrine VEGF promoting glycolysis dependent-survival of colorectal cancer cells in the circulation
Source: J Exp Clin Cancer Res. 2020 Jan 30;39:25. doi: 10.1186/s13046-020-1533-0 (PMC6993408; doi:10.1186/s13046-020-1533-0)
Supplement: Supplementary file 4 — Additional file 4: Table S2. Demographics and clinical characteristics of 141 cases of CRC patients. [file 13046_2020_1533_MOESM4_ESM.pdf]

Table S2. Demographics and Clinical Characteristics of 141 cases of CRC patients

Characteristic			Non-hypertension		Hypertension		*P* value
	n=141	%	n=114	%	n=27	%	
**Sex**							
male	85	60.3	68	59.6	17	63.0	0.752
female	56	39.7	46	40.4	10	37.0	
**Age (years)**							
≤ 60	93	34.0	80	70.2	13	51.9	0.030
>60	48	66.0	34	29.8	14	48.1	
**BMI index**							
<18.5	16	11.3	15	13.2	1	3.7	0.338
18.5-24.9	107	75.9	84	73.7	23	85.2	
>=25	18	12.8	15	13.2	3	11.1	
**Tumour site**							
left colon	47	33.3	37	32.5	10	37.0	0.528
right colon	39	27.7	30	26.3	9	33.3	
rectum	55	39.0	47	41.2	8	29.6	
**Tumour differentiation**							
Poorly	16	11.3	13	11.4	3	11.1	0.827
Moderately	88	62.4	70	61.4	18	66.7	
Well	19	13.5	15	13.2	4	14.8	
-	18	12.8	16	14.0	2	7.4	
**TNM stage**							
I	1	0.7	1	0.9	0	0.0	0.926
II	17	12.1	13	11.4	4	14.8	
III	22	15.6	18	15.8	4	14.8	
IV	101	71.6	82	71.9	19	70.4	
**Lymph node metastasis**							
positive	72	51.1	58	50.9	14	51.9	0.927
negative	69	48.9	56	49.1	13	48.1	
**Liver metastasis**							
positive	68	48.2	56	49.1	12	44.4	0.662
negative	73	51.8	58	50.9	15	55.6	
**Lung metastasis**							
positive	39	27.7	30	26.3	9	33.3	0.464

negative	102	72.3	84	73.7	18	66.7	
**Organ metastasis(Others)**							
positive	23	16.3	19	16.7	4	14.8	0.815
negative	118	83.7	95	83.3	23	85.2	
**Gene mutation**							
KRAS	22	15.6	17	14.9	5	18.5	0.100
BRAF	3	2.1	1	0.9	2	7.4	
other	2	1.4	1	0.9	1	3.7	
-	114	80.9	95	83.3	19	70.4	

Note. The correlation was examined using Chi-square test.
